# Computer-Based Cognitive Training for Executive Functions after Stroke: A Systematic Review

**DOI:** 10.3389/fnhum.2016.00150

**Published:** 2016-04-20

**Authors:** Renate M. van de Ven, Jaap M. J. Murre, Dick J. Veltman, Ben A. Schmand

**Affiliations:** ^1^Department of Psychology, Brain and Cognition, University of AmsterdamAmsterdam, Netherlands; ^2^Department of Psychiatry, VU University Medical CenterAmsterdam, Netherlands; ^3^Department of Medical Psychology, Academic Medical Centre, University of AmsterdamNetherlands

**Keywords:** working memory, attention, restitution, retraining, acquired brain injury, brain training, executive functions, computer-based

## Abstract

**Background:** Stroke commonly results in cognitive impairments in working memory, attention, and executive function, which may be restored with appropriate training programs. Our aim was to systematically review the evidence for computer-based cognitive training of executive dysfunctions.

**Methods:** Studies were included if they concerned adults who had suffered stroke or other types of acquired brain injury, if the intervention was computer training of executive functions, and if the outcome was related to executive functioning. We searched in MEDLINE, PsycINFO, Web of Science, and The Cochrane Library. Study quality was evaluated based on the CONSORT Statement. Treatment effect was evaluated based on differences compared to pre-treatment and/or to a control group.

**Results:** Twenty studies were included. Two were randomized controlled trials that used an active control group. The other studies included multiple baselines, a passive control group, or were uncontrolled. Improvements were observed in tasks similar to the training (near transfer) and in tasks dissimilar to the training (far transfer). However, these effects were not larger in trained than in active control groups. Two studies evaluated neural effects and found changes in both functional and structural connectivity. Most studies suffered from methodological limitations (e.g., lack of an active control group and no adjustment for multiple testing) hampering differentiation of training effects from spontaneous recovery, retest effects, and placebo effects.

**Conclusions:** The positive findings of most studies, including neural changes, warrant continuation of research in this field, but only if its methodological limitations are addressed.

## Introduction

Stroke, resulting from brain hemorrhage or infarction, commonly results in cognitive impairments such as aphasia, neglect, reduced processing speed, impaired attention, and executive dysfunction (e.g., Cumming et al., 2013). Even though cognition can improve during the first year after stroke (Desmond et al., 1996; Tham et al., 2002; del Ser et al., 2005), cognitive impairment frequently persists long after. More than 60% of stroke survivors still reported mild to severe cognitive impairment up to 10 years after stroke (Maaijwee et al., 2014; Middleton et al., 2014). Furthermore, cognitive impairments continue to deteriorate in 11% of stroke survivors during the first year after stroke (Tham et al., 2002). Therefore, rehabilitation efforts to ameliorate these cognitive impairments are essential.

Guidelines for neurorehabilitation are mainly focused on compensational strategy training (Cicerone et al., 2011). These strategies do not aim to restore brain functions (i.e., restitution), but aim to compensate for the lost function by using remaining intact functions. In this approach, residual plasticity of the brain throughout adulthood, which may enable restitution of the impaired function, is ignored (e.g., Kelly et al., 2006; Takeuchi and Izumi, 2015).

Robertson and Murre (1999) postulated that depending on the amount of remaining connectivity, different types of intervention are needed, notably restitution or compensation. Mildly damaged brain networks might reconnect by everyday life activities, and no special intervention is necessary. Severely affected brain networks may not be able to reconnect at all. Therefore, in severe cases compensational interventions are required that make use of preserved networks. For moderately affected networks, restitution-based interventions may be needed to stimulate the relevant parts of the impaired network.

Restitution focused treatments commonly consist of massed frequent repetition or stimulation of the affected function (Hamzei et al., 2006). They have proven to be effective in the domains of language, motor function, and vision (e.g., Kurland et al., 2010; Thrane et al., 2014). For other cognitive domains, such as attention and executive function, restitution training may consist of, for example, training reaction speed. Conversely, compensation interventions may consist of, for example, time management training to teach the patient to take more time for task execution. One type of restitution-based interventions use computer tasks aimed at training of damaged networks.

To date it is not yet clear whether restitution-based computer training can improve attention, working memory, and executive functions. In healthy adults, training effects have been contradictory (e.g., Owen et al., 2010; Anguera et al., 2013; Corbett et al., 2015), but a recent meta-analysis concluded that cognition can be improved (Toril et al., 2014). A systematic review of 10 studies in stroke patients concluded that restitution- and compensation-based interventions improved executive functions (Poulin et al., 2012). Even though the review by Poulin et al. did not only focus on restitution-based computerized training programs, their review does provide ground to further evaluate these restitution-based training programs.

This systematic review provides an overview of the evidence concerning the effects of computer-based restitution rehabilitation after stroke and other acquired brain damage to restore executive functioning. The term executive function includes a spectrum of cognitive functions, all revolving around control of one's behavior. This includes mental set shifting (i.e., changing from one set of task rules to another), information updating, and inhibition of prepotent but inappropriate responses (Miyake et al., 2000). For this review we considered working memory and divided (or selective) attention as part of the executive domain. Training programs that only focused on vigilance, tonic alertness, and sustained attention without any divided or selective attention tasks were not included.

## Methods

### Search strategies

We performed this systematic review according to the Preferred Reporting Items for Systematic Reviews and Meta-Analyses (PRISMA, Moher et al., 2009) statement. We searched in MEDLINE, PsycINFO, Web of Science, and The Cochrane Library. The search terms entered were a combination of three search areas that defined (1) the population as adults who had suffered a stroke or acquired brain injury, (2) the intervention as executive function computer training, and (3) the outcome as executive functioning. The complete search strategy can be found in Supplementary Material 1.

### Inclusion and exclusion criteria

We considered articles in English, limited to humans, and published before the 12th of May 2015. Included participants were adults who had suffered stroke or other acquired brain injury. Computer training had to be the main intervention with a focus to improve working memory, attention related to executive functioning, or executive functioning.

**Figure 1:**
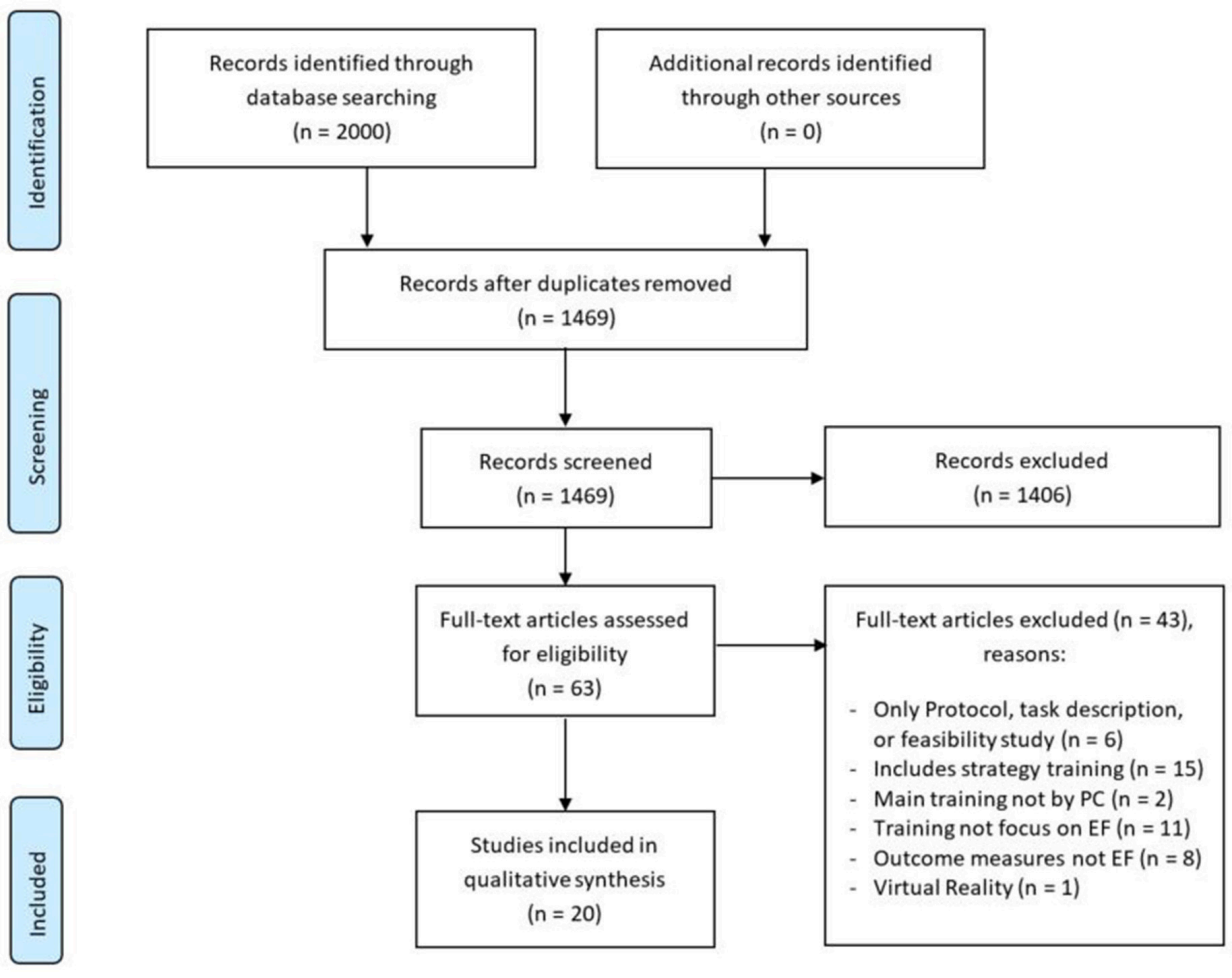
**PRISMA flow diagram (Moher et al., 2009) of study identification process**.

Studies of strategy education or virtual reality training were excluded. Study protocols and dissertations were not considered. The selection of studies was first based on screening of title and abstract, followed by reading of the full text of the remaining studies (see flowchart in Figure 1). When in doubt, selection was discussed until consensus was reached.

### Rating of methodological quality

The quality of the included studies was evaluated based on recommendations for reporting trials of the Consolidated Standards of Reporting Trials (CONSORT) statement (see Table 1). For each study, we also extracted the authors; year of publication; population; control group; training and its focus, duration, and setting; outcome measures and their significance level; the presence of adjustments for multiple testing; whether performance on training was related to outcome measures; use of ecologically valid measures; and potential conflicts of interest (see Tables 2–**4**). Treatment effect was evaluated based on statistically significant differences compared to pre-treatment and/or to a control group. Whenever adjustment for multiple testing was not performed and *p*-values were provided, we adjusted the reported *p*-values with Bonferroni-Holm correction. Similarly, for studies that did adjust but provided sufficient information to calculate the unadjusted *p*-value, Tables 2–**4** show tasks that would be significant *without* the adjustment. Due to the heterogeneity of the outcome measures, it was not possible to perform a meta-analysis.

**Table 1:** **Study quality based on the CONSORT statement**.

	**Study**	**Working memory**				**Attention**								**Multi modal**							**Imaging**
		**Akerlund et al., 2013**	**Björkdahl et al., 2013**	**Lundqvist et al., 2010**	**Westerberg et al., 2007**	**Gauggel and Niemann, 1996**	**Hauke et al., 2011**	**Ponsford and Kinsella, 1988**	**Prokopenko et al., 2013**	**Sturm et al., 1997**	**Sturm et al., 2003**	**van Vleet et al., 2015**	**Zickefoose et al., 2013**	**Chen et al., 1997**	**De Luca et al., 2014**	**Fernandez et al., 2012**	**Gray et al., 1992**	**Ruff et al., 1994**	**Spikman et al., 2010**	**Lin et al., 2014**	**Nordvik et al., 2012**
	**Design**	**B**	**B**	**B**	**B**	**C**	**C**	**C**	**B**	**G**	**C**	**E**	**C**	**F**	**B**	**G**	**A**	**D**	**A**	**B**	**C**
	**Total score**	**11**	**9.5**	**9.5**	**11.5**	**7.5**	**8.5**	**8.5**	**10.5**	**8.5**	**9.5**	**7**	**9.5**	**7**	**9.5**	**8.5**	**8.5**	**7.5**	**9.5**	**9**	**7**
Methods	Eligibility criteria participants	+	+	+	+	+	−	+	+	+	+	+	+	+	+	+	+	+	+	+	−
	Setting data collection	+	+	+	+	−	±	±	+	+	+	+	±	+	±	+	±	±	±	±	±
	Intervention description	+	+	+	+	±	+	+	+	+	+	±	+	−	±	±	+	±	±	±	±
	Outcome description	+	+	+	±	±	±	±	±	+	+	±	+	±	±	+	±	+	+	±	±
	Determination of N	−	−	−	−	−	−	−	−	−	−	−	−	−	−	−	−	−	−	−	−
	Randomization	+	+	+	+	−	n.a.	+	+	−	±	−	+	−	+	−	+	+	+	+	n.a.
	Blinding	−	−	−	−	−	n.a.	−	±	−	−	−	−	−	−	−	−	−	±	±	n.a.
	Statistical methods used	+	+	+	+	+	+	+	+	+	+	−	±	+	+	+	+	±	+	+	±
Results	N per group	+	+	+	+	+	+	+	+	+	+	+	+	+	+	+	+	+	+	±	+
	Attrition per group	±	−	+	+	+	+	±	+	±	±	+	+	n.a.	+	±	+	±	±	−	+
	Recruitment dates	+	+	−	−	−	+	−	−	±	−	−	−	+	+	+	−	−	−	+	−
	Termination reason	−	−	−	−	−	−	−	−	−	−	−	−	n.a.	−	−	−	−	−	−	−
	Baseline characteristics table	+	−	−	+	+	+	+	+	−	+	+	+	−	+	−	+	−	+	+	+
	N per analysis	+	±	+	+	+	+	±	+	+	+	+	+	+	±	−	−	+	+	+	+
	Extend of results reports	±	+	±	+	±	±	±	±	±	±	−	±	±	±	±	±	±	±	±	+
	Harms	−	−	−	+	−	−	−	−	−	−	−	−	−	−	+	−	−	−	−	−

*Akerlund et al. (2013) and Björkdahl et al. (2013) used the same dataset; A, RCT with active control group (CG); B, RCT with passive CG; C, single case, multiple baseline; D, uncontrolled, multiple baseline; E, single case, matched controlled; F, Retrospective, case matched controlled; G, uncontrolled, pre-post; +, reported; ±, partially sufficient; -, not sufficient; n.a., not applicable*.

**Table 2:** **An overview of working memory training studies**.

**Study (focus)**	**Population**	**Sample size**	**Training (focus)**	**Duration training (actual mean)**	**Setting**	**Outcome measures**	**Significance of comparisons of pre-training (t_0_), post-training (t_1_), and follow-up (t_2_)**							**Multiple testing adjusted**	**Ecological valid measure**	**Train. improv. Evaluated**	**Reported conflicts of interest**
							**Within Ss**				**Between Ss**						
							**t_1_–t_0_**		**t_2_–t_0_**	**t_2_–t_1_**	**t_1_–t_0_**	**t_2_–t_0_**	**t_2_–t_1_**				
							**CG**	**IG**									
Akerlund et al., 2013 (WM, cognitive function, psychological health)	ABI, post-acute - chronic	*N* = 38: IG = 20 CAU = 18, cross-over	QM, Cogmed (WM)	30–45 min, 5 d/w, 5 w = 15,6 h (unkn.)	Rehab.; Coach feed-back 1/w	Dig Span	−	+	+	−	−	±	−	No	Yes, question-naire	No	None
						Dig Span rev.	−	+	+	−	−	+	−				
						Span board	−	−	−	−	−	−	−				
						Span board rev.	+	−	−	−	−	−	−				
						WM composite	+	+	+	−	−	−	−				
						BNIS	−	+	+	−	±	−	−				
						DEX	−	−	−	−	−	−	−				
						HADS anx.	−	−	−	−	−	−	−				
						HADS depr.	−	−	+	−	−	−	−				
Björkdahl et al., 2013 (WM, ADL)	See Akerlund et al.	See Akerlund et al.	QM, Cogmed (WM)	See Akerlund et al.	See Akerl. et al.	AMPS-motor	−	−	±	±	×	−	×	No	Yes, question-naires	No	None
						AMPS-process	−	−	−	−	×	−	×				
						RBMT-II	−	−	−	−	×	−	×				
						FIS	−	±	−	−	×	−	×				
						WM quest.	−	×	+	×	×	±	×				
Lundqvist et al., 2010 (complex WM, QoL and health)	ABI, chronic (≥ 1 y post onset)	*N* = 21: IG = 10 pCG = 11, cross-over	QM, Cogmed (WM)	45–60 min, 5 d/w, 5 w = 22 h [actual train sessions = 21–25 d (*M* = 23.2; SD = 1.6)]	Rehab.; Coach feed-back 1/w	PASAT	−	+^a^	+	.	.	.	.	Yes, Bonferroni correct. for tasks not for question-naires	Yes, question-naires	Yes, but not related to outcome measures	None
						LSPAN	−	+^a^	+	.	.	.	.				
						Picture Span	−	+^a^	+	.	.	.	.				
						Block Span forw.	−	+^a^	+	.	.	.	.				
						Block Span rev.	−	+^a^	+	.	.	.	.				
						CWIT inh./swtch.	−	+^a^	+	.	.	.	.				
						COPM - perform.	.	.	+	.	.	.	.				
						COPM - satisfy	.	.	+	.	.	.	.				
						EQ-5D	.	.	−	.	.	.	.				
						Health VAS	.	.	±	.	.	.	.				
Westerberg et al., 2007 (visuo-spatial and auditory WM)	first stroke, chronic (1–3 y post onset)	*N* = 18: IG = 9 pCG = 9	QM, Cogmed (WM)	40 min, 5 d/w, 5 w = 16.7 h [actual train sessions *M* = 23 d (SD = 2.2)]	Home; Coach feed-back 1/w by phone	Digit span Span board Word list learning Word list learning DR	.	.	.	.	+	.	.	No	Yes, question-naire	Yes, but not related to outcome measures	Yes, stock holders in company of software used
							.	.	.	.	±	.	.				
							.	.	.	.	−	.	.				
							.	.	.	.	−	.	.				
						PASAT	.	.	.	.	+	.	.				
						Ruff 2 & 7 SAT	.	.	.	.	+	.	.				
						Stroop - time	.	.	.	.	−	.	.				
						Stroop - raw	.	.	.	.	−	.	.				
						Raven's PM	.	.	.	.	−	.	.				
						CFQ	.	.	.	.	+	.	.				

*+, significant (sign.) and survives adjustment for multiple testing (Bonferroni-Holm adjustment is based on the number of outcome measures used at that time-point); ±, sign. but doesn't survive adjustment; -, not sign.; ×, not included in analysis;., not mentioned in methods; a, post measure was 4 weeks after training completion*.

*Train. improv., training improvements; Ss, subjects; IG, intervention group; (p)CG, (passive) control group; CAU, care as usual; N, sample size; y, years; w, weeks; d/w, days per week; h, hours; min, minutes; WM, working memory; ADL, activities of daily living; QoL, quality of life; ABI, acquired brain injury; unkn., unknown; M, mean; SD, standard deviation; rehab., rehabilitation center; correct., correction*.

*Abbreviations neuropsychological tests: Rev., reversed; BNIS, Barrow Neurological Institute Screen for Higher Cerebral Functions; DEX, Dysexecutive Questionnaire; HADS, Hospital Anxiety and Depression Scale; anx., anxiety; depr., depression; AMPS, Assessment of Motor and Process Skills; RBMT, Rivermead Behavioral Memory Test; FIS, Fatigue Impact Scale; quest., questionnaire; PASAT, Paced Auditory Serial Attention Test; LSPAN, Listening Span Test; forw., forward; CWIT inh./swtch., Color Word Interference Test inhibition/switching; COPM, Canadian Occupational Performance Measure; perform., performance; EQ-5D, EuroQol 5D; VAS, visual analog scale; DR, delayed recall; SAT, Selective Attention Test; PM, progressive matrices; CFQ, Cognitive Failures Questionnaire*.

## Results

We reviewed 1469 titles and abstracts; 63 studies were reviewed based on full-text. Twenty studies satisfied inclusion and exclusion criteria for this systematic review (see Figure 1). An overview of the data extracted is listed in Tables 2–**4**.

The included studies consisted of nine randomized controlled trials (RCT), six single case studies, four uncontrolled trials of which two used multiple baselines (i.e., multiple measurement time-points before training onset), and one retrospective study. Two studies used an active control group (i.e., the control group received an alternative, but supposedly ineffective training), and seven studies used a passive control group (i.e., the control group did not receive anything in addition to care as usual). The median sample size was 32 (range: 1–75). Two studies had a single subject design (i.e., *n* = 1). Two studies used the same sample (Akerlund et al., 2013; Björkdahl et al., 2013). Five studies included post-acute patients, six included chronic patients, and nine included a combination of both.

Scores on the selected CONSORT statement criteria ranged from 7 to 11.5 out of maximum 16 (see Table 1). Setting of training (e.g., given at home or in the rehabilitation center with or without supervision) was described in 11 studies. In all but two studies (Chen et al., 1997; De Luca et al., 2014) reports of training duration included the scheduled number of sessions per week. The median *planned* number of hours of training was 15.6 (range: 4.5–60). Only three studies included the *actual* number of training hours performed by the participants (Gray et al., 1992; Westerberg et al., 2007; Lundqvist et al., 2010).

Blinding of assessors was done in three studies, but the participants were never blinded. Description of outcome measures commonly included the name of the task, but not which specific task parameter was used (e.g., raw scores or scaled scores, response times or number of errors). One study did not use statistical methods to evaluate its results (van Vleet et al., 2015). Potential harms of the training were evaluated in two studies. One study reported no harms (Westerberg et al., 2007); the other reported mental fatigue, headache, and eye irritation (Fernandez et al., 2012).

Four studies adjusted for multiple statistical testing (Chen et al., 1997; Sturm et al., 1997, 2003; Spikman et al., 2010), and one corrected part of the statistical tests (Lundqvist et al., 2010). None of the studies correlated improvements on outcome measures with progression of performance during the training. Four studies examined performance on the training tasks itself, which improved in all studies (Westerberg et al., 2007; Lundqvist et al., 2010; Zickefoose et al., 2013; van Vleet et al., 2015). Two studies reported conflicts of interest (Ruff et al., 1994; Westerberg et al., 2007), six studies reported no conflicts of interest, and 12 studies did not report on this. The extracted studies evaluated working memory training, attention training, or both. We will now discuss the evidence of these training programs in more detail.

### Working memory training

Working memory is the storage of information for a short period of time such that it can be manipulated (Baddeley, 1992). It is important for many other cognitive functions such as planning, problem solving, and learning. It is crucial for everyday functioning, which is one of the reasons that it is the focus of many training studies (Westerberg et al., 2007; Lundqvist et al., 2010; Akerlund et al., 2013; Björkdahl et al., 2013). The most common computerized working memory training currently used is Cogmed QM (from Cogmed Systems AB, Stockholm, Sweden; now published by Pearson Assessment and Information B.V.).

#### Cogmed training

The Cogmed training consists of five 30–40 min sessions per week during 5 weeks. Thus, a total of about 15 h of training is provided. It includes both audio (verbal) and visual (visuospatial) working memory tasks, which always require a motor response. Task difficulty is adapted to the performance of the trainee, and positive feedback is given immediately. It is a computer-based program that can either be done at the rehabilitation center (Lundqvist et al., 2010; Akerlund et al., 2013; Björkdahl et al., 2013) or at home (Westerberg et al., 2007). A coach monitors the progression of the trainee and contacts the trainee once per week to provide individual feedback. A detailed description of each task used in the training can be found elsewhere (Westerberg et al., 2007).

#### Objective improvements of working memory

The training resulted in improvements on most objective working memory tasks used (Westerberg et al., 2007; Lundqvist et al., 2010; Akerlund et al., 2013) and the effects remained stable during three (Akerlund et al., 2013) or 5 months after training completion (Lundqvist et al., 2010; see Table 2 for an overview). The tasks used to evaluate the training were all fairly similar to the training tasks and included verbal and visuospatial tasks, but some tasks were dissimilar to the training. This is important, because improvements only on tasks that are similar to the training (i.e., near transfer effect) are less likely to contribute to improvements in daily living than improvements that also generalize to tasks that are dissimilar to the training (i.e., far transfer effects). Far transfer was observed for complex working memory tasks that involved more than just remembering the stimuli (Lundqvist et al., 2010). These improvements in the intervention group (*n* = 21) were not observed in the passive control group (*n* = 11), but the two groups were not directly compared. The improved performance of one of these complex working memory tasks remained significant 5 months after training completion.

#### Objective improvements in untrained cognitive tasks

Objective improvements were not only observed on working memory tasks. General cognitive performance, as measured by an elaborate screening, significantly improved after training, also in comparison to the control group (Akerlund et al., 2013).

Improvements in other cognitive domains were mixed. Attention, which is closely related to working memory, also benefited from working memory training (Westerberg et al., 2007). Conversely, performance on a non-trained reasoning task did not improve significantly more than in the control group (Westerberg et al., 2007). The effect of the working memory training on inhibition appears somewhat inconclusive. Improvement on the Stroop color-word interference task was not significantly greater than in the control group (Westerberg et al., 2007). In another study, however, scores on the inhibition and switching condition of the slightly different Color Word Interference Test (CWIT) significantly improved after the training and remained stable 20 weeks after training completion (Lundqvist et al., 2010). This task seems to involve more working memory than the Stroop task, as it requires not only inhibiting a preferred response, but also switching between two task sets (i.e., mentioning the color of the ink vs. mentioning the letters of the word). This may explain why improvement of working memory could benefit CWIT performance and, thus, may not reflect improved inhibition *per se*.

#### Subjective improvements

Working memory training also seems to improve subjective functioning in daily life. Improvements were seen in subjective ratings of working memory and in the effects of fatigue on daily living (Björkdahl et al., 2013), subjective cognitive functioning (Westerberg et al., 2007), and (satisfaction with) occupational performance (Lundqvist et al., 2010). It did not specifically improve subjective executive functioning (Akerlund et al., 2013). Effects of the training on health related quality of life were inconsistent as a significant improvement was only found for one of two questionnaires (Lundqvist et al., 2010).

However, all these studies used a control group that received either no training (Westerberg et al., 2007; Lundqvist et al., 2010) or care as usual (Akerlund et al., 2013; Björkdahl et al., 2013). Factors such as social contact or placebo effects may have accounted for the reported results. Nevertheless, Westerberg and colleagues reported that the effect of the training on the subjective measure of cognitive functioning was mostly in items related to attention and not in more general items. This suggests that it was a real training effect. Future studies should include an active control group that receives a mock training to control for placebo effects.

The question is, however, whether a placebo effect should be seen as irrelevant. The subjective experience of participants is important as this may improve their mood and self-confidence. Indeed, mood seemed to improve after working memory training (Akerlund et al., 2013). Furthermore, as Lundqvist suggested, following the structured training program may prepare individuals for returning back to work as they need to adhere to appointments and schedules in both instances.

#### Limitations of working memory training studies

Apart from the lack of appropriate control groups, another limitation of most of these studies is that they did not adjust for multiple statistical testing (Westerberg et al., 2007; Akerlund et al., 2013; Björkdahl et al., 2013), or only for part of the statistical tests (Lundqvist et al., 2010). An overview of which tasks would survive adjustment for multiple testing can be found in Table 2.

If multiple testing and comparisons with appropriate control groups were taken into account, some effects would disappear. From the objective working memory measures, only digit span backwards appeared to be significantly improved immediately after training (Westerberg et al., 2007; Akerlund et al., 2013) and at 3 months follow-up (Akerlund et al., 2013). The objective improvements of attention would remain significant and thus seem promising (Westerberg et al., 2007). Of the subjective measures, only subjective cognitive improvement tended to remain significant (Westerberg et al., 2007). In the study by Björkdahl et al. (2013) none of the between-group comparisons of subjective measures remained significant after adjusting for multiple testing, suggesting that these effects were not robust.

In two out of three studies there was no effect of the training on the visuospatial working memory task after adjustment for multiple testing (Westerberg et al., 2007; Akerlund et al., 2013). The visuospatial tasks used in the training may not have been sufficiently challenging to elicit transfer effects.

Lundqvist et al. (2010) and Westerberg et al. (2007) reported improved performance on training tasks. If improvements in cognition are due to the training, there needs to be a substantial correlation between the two. However, none of the studies related the improvements of the outcome measures to the improvement observed during the training.

#### Conclusion of working memory training studies

In sum, there is preliminary evidence that Cogmed can improve performance on tasks that are similar to the training (near transfer) and tasks that are dissimilar to the training (far transfer). This is the case for both objective working memory and attention. It also seems to improve subjective cognitive functioning. Moreover, the effect of the training has been shown for verbal working memory but not for visual working memory. Nevertheless, all studies described so far suffered from methodological limitations, to which we will return in the discussion section.

### Attention training

#### AixTent training

Training programs aimed at improving attention are more diverse than those aimed at working memory (see Table 3A for an overview of attention studies with double baseline and Table 3B for studies with single baseline). One commonly used training is AixTent, which consists of separate training modules that can be combined. The modules focus on phasic alertness, vigilance, selective attention, or divided attention. Responses can be given with two response keys that can also be operated with only one hand. All tasks were designed to be game-like, and task difficulty is automatically adapted to the performance of the participant. Feedback is given during and at the end of a training session.

**Table 3A:** **An overview of attention training studies with double baseline measurement**.

**Study**	**Population**	**Sample size**	**Training (focus)**	**Duration training (actual mean)**	**Setting**	**Outcome measures**	**Significance of comparisons of pre-training (t_0_), second baseline (t_0b_), post-training (t_1_), and follow-up (t_2_)**							**Multiple testing adjusted**	**Ecological valid measure**	**Train. improv. Evaluated**	**Reported conflicts of interest**
							**Within Ss**				**Between Ss**						
							**t_0b_–t_0_**	**t_1_–t_0_**	**t_2_–t_0_**	**t_2_–t_1_**	**t_1_–t_0_**	**t_2_–t_0_**	**t_2_–t_1_**				
Gauggel et al., 1996	ABI, post-acute - chronic (4–16 m post onset) with deficit in ≥2/3 NPA att. tasks	*N* = 4	Hierarchical (alertness, RT, vigilance, suppress interference, selective and divided att.)	30–40 min, 5 d/w, 2–4 w = 12.5 h (unkn.)	Unkn.	Attention:								No	No, only global question-naire	No	Not reported
						WDG	_*_^a^	−	.	.	.	.	.				
						Numb. connect.	_*_^a^	_*_^a^	.	.	.	.	.				
						D2	−	_*_^a^	.	.	.	.	.				
						WMS:											
						Logical mem.	−	−	.	.	.	.	.				
						Assoc. learning	−	−	.	.	.	.	.				
						CFT - Rey	−	−	.	.	.	.	.				
						LPS (IQ)	−	−	.	.	.	.	.				
						Sat. w Life Scale	_*_^a^	−	.	.	.	.	.				
						CES-D	−	−	.	.	.	.	.				
Hauke et al., 2011	Brainstem encephalitis, chronic (4.5 y post onset)	*N* = 1	CogniPlus (alertness)	45 min, 5 d/w, 3 w = 11 h (unkn.)	Unkn.	Intrinsic alert.	−	^*^	.	-	.	.	.	No	Yes, question-naire of att. deficit	No	Not reported
						Focused att.	_*_	_*_	.	- -	.	.	.				
						Vigilance	_*_	_*_	.	-	.	.	.				
						Divided att.:											
						Audio	_*_	_*_	.	- -	.	.	.				
						Visual	_*_	_*_	.	-	.	.	.				
						subj. att. (FEDA)	.	_*_	.	-	.	.	.				
Ponsford et al., 1988	Very severe HI, post-acute (<9 m post onset) with deficit in SoP	*N* = 15 (10 comple-ted)	Unkn. (speed of visual RT, visual search, selective att.)	15 times 30 min, 3 w = 7.5 h (unkn.)	Rehab.?	Four-choice RT	↑	−	↑	.	.	.	.	No	Yes, clerical task	No	Not reported
						SDMT	↑	−	↑	.	.	.	.				
						2-letter cancel.	↑	−	↑	.	.	.	.				
						Similarities	.	−	^*^	.	.	.	.				
						subj. Att. Beh.	↑	−	^*^	.	.	.	.				
						Clerical task	.	−	−	.	.	.	.				
																	
Sturm et al., 2003	ABI, post-acute - chronic (3 m - 13 y post onset) with att. deficits in ≥2 att. domains	*N* = 33: 9: alertness 7: vigilance 11: selective att. 6: divided att.	AIXTENT (either alertness, vigilance, selective, or divided att.) + CAU (but no other att. therapies)	14 times 1 h = 14 h (unkn.)	Rehab.	TAP:								Yes, Bonfer-roni correct.	No	No	Not reported
						Alertness:											
						Phasic	^-*^	+^b^	.	.	.	.	.				
						Intrinsic	^-*^	+^b^	.	.	.	.	.				
						Vigilance:											
						Error	^-*^	^-*^	.	.	.	.	.				
						Omission	^-*^	+^b^	.	.	.	.	.				
						Selective att.:											
						Error	^-*^	^-*^	.	.	.	.	.				
						RT	^-*^	^-*^	.	.	.	.	.				
						Divided att.:											
						Omission	+	+^b^	.	.	.	.	.				

+, significant (sign.) and survives adjustment for multiple testing (Bonferroni-Holm adjustment is based on the number of outcome measures used at that time-point); -, not sign.; ^*^, sign. but unknown whether it would survive adjustment; -^*^, not sign. but unknown whether it would be sign. without adjustment; - -, sign; decrease ↑, improvement but not statistically tested;., not mentioned in methods; a, max 1/4 of patients sign. improved; b, max 1/4 training programs resulted in sign. improvement.

*Train. improv., training improvements; Ss, subjects; N, sample size; y, years; m, months; w, weeks; d/w, days per week; h, hours; min, minutes; ABI, acquired brain injury; NPA, Neuropsychological assessment; att., attention; HI, head injury; SoP, speed of processing; RT, reaction time; unkn., unknown; CAU, care as usual; rehab., rehabilitation center; correct., correction*.

*Abbreviations neuropsychological tests: WDG, Wiener Determination Apparatus; Numb. connect., Number Connection Test; WMS, Wechsler Memory Scale; mem., memory; Assoc., Associated; CFT, Complex Figure Test; LPS, Leistungsprüfsystem; Sat. w Life Scale, Satisfaction with Life Scale; CES-D, Center for Epidemiologic Studies Depression Scale; alert., alertness; subj. att. (FEDA), subjective attention (Fragebogen erlebter Defizite der Aufmerksamkeit); SDMT, Symbol Digit Modalities Test; 2-letter cancel., Two-letter cancellation task; subj. Att. Beh., Rating Scale of Attentional Behaviors; TAP, Test of Attentional Performance*.

**Table 3B:** **An overview of attention training studies with single baseline measurement**.

**Study**	**Population**	**Sample size**	**Training (focus)**	**Duration training (actual mean)**	**Setting**	**Outcome measures**	**Significance of comparisons of pre-training (t_0_), post-training (t_1_), and follow-up (t_2_)**							**Multiple testing adjusted**	**Ecological valid measure**	**Train. improv. Evaluated**	**Reported conflicts of interest**
							**Within Ss**				**Between Ss**						
							**t_1_–t_0_**		**t_2_–t_0_**	**t_2_–t_1_**	**t_1_–t_0_**	**t_2_–t_0_**	**t_2_–t_1_**				
							**CG**	**IG**									
Prokopenko et al., 2013	Stroke, acute—post-acute (< 2 w) with mild cognitive impairments to mild dementia	*N* = 43: IG = 24 CAU = 19	Neuropsychological computer training (sustained, selective, divided, and alternating att.)	30 min, 7 d/w, 2 w = 15 h (unkn.)	Rehab.	Schulte's tables	±	±	.	.	±	.	.	No	Yes, question-naires but not specific about EF	No	None
						Clock drawing	−	±	.	.	±	.	.				
						MMSE	−	±	.	.	−	.	.				
						MoCA	±	±	.	.	−	.	.				
						FAB	−	±	.	.	±	.	.				
						HADS anx.	−	−	.	.	×	.	.				
						HADS depr.	−	−	.	.	×	.	.				
						IADL	−	−	.	.	×	.	.				
						SS-QOL-2	−	−	.	.	×	.	.				
						PGIS	=	↑	.	.	×	.	.				
						CGIS	×	×	.	.	×	.	.				
van Vleet et al., 2015	Mild TBI, chronic (2–12 y) with Executive dysfunc.	*N* = 5: IG = 3 CG = 2 (contact matched)	TAPAT (tonic and phasic alertness)	9 times 36 min, 3 w = 4.5 h (unkn.)	Home?	EF:	Ratio pts clinically sign. improved:							N.a., no stat. analyses	No	Yes, but not related to outcome measures	Not reported
						ACT-18s	0/2	2/3	.	.	.	.	.				
						ACT-36s	0/2	0/3	.	.	.	.	.				
						TMT-B	1/2	2/3	.	.	.	.	.				
						verb. Fluency	0/2	2/3	.	.	.	.	.				
						LNS	0/2	2/3	.	.	.	.	.				
						Att. blink	0/2	3/3	.	.	.	.	.				
						PCL:											
						Concentration	×	2/3	.	.	.	.	.				
						Hyper-vigilance	×	1/3									
						Training – acc.	0/2	3/3	.	.	.	.	.				
						Training - RT	0/2	1/3	.	.	.	.	.				
Sturm et al., 1997	Unilateral stroke, post-acute—chronic (2 m-3 y post stroke) with att. deficits in ≥2 att. domains	*N* = 38: No control	AIXTENT (either alertness, vigilance, selective, or divided att.)	14 times 1 h = 14 h (unkn.)	Rehab.	TAP:								Yes, Bonfer-roni correct.	No	No	Not reported
						Alertness:											
						no warning	.	+^a^	.	.	.	.	.				
						warning	.	^-*^	.	.	.	.	.				
						Vigilance - hits	.	+^a^	.	.	.	.	.				
						Vigilance - RT	.	^-*^	.	.	.	.	.				
						Selective att.:											
						Error	.	−	.	.	.	.	.				
						RT	.	+^a^	.	.	.	.	.				
						Divided att.:											
						Error	.	+^a^	.	.	.	.	.				
						RT	.	+^a^	.	.	.	.	.				
Zickefoose et al., 2013	Severe TBI, chronic (≥3 y post injury)	*N* = 4: A-B-A-C-A design; B first: *n* = 2, C first: *n* = 2	B: Hierarchically-based Attention Process Training (att.; 4 w) C: Lumosity (att.; 4 w)	Per training 20 times 30 min in 1 m = 20 h (unkn.)	Unknw. (researcher was present)	Training	.	^*^	.	.	.	.	.	No	No	Yes, but not related to outcome measures	None
						TEA	.	?	.	.	.	.	.				
						Att. probe	.	?	.	.	.	.	.				

+, significant (sign.) and survives adjustment for multiple testing (Bonferroni-Holm adjustment is based on the number of outcome measures used at that time-point); ±, sign. but doesn't survive adjustment; -, not sign.; ^*^, sign. but unknown whether it would survive adjustment; -^*^, not sign. but unknown whether it would be sign. without adjustment; ↑, improvement but not statistically tested; =, no improvement but not statistically tested; ?, not conclusive; ×, not included in analysis;., not mentioned in methods; a, max 2/4 training programs resulted in sign. improvement.

*Train. improv., training improvements; Ss, subjects; IG, intervention group; CG, control group; CAU, care as usual; N, sample size; y, years; m, months; w, weeks; d/w, days per week; h, hours; min, minutes; TBI, traumatic brain injury; dysfunc., dysfunction; att., attention; unkn., unknown; rehab., rehabilitation center; n.a., not applicable; stat., statistical; correct., correction; EF, executive functioning*.

*Abbreviations neuropsychological tests: MMSE, Mini-Mental State Examination; MoCA, Montreal Cognitive Assessment; FAB, Frontal Assessment Battery; HADS, Hospital Anxiety and Depression Scale; anx., anxiety; depr., depression; IADL, Instrumental Activities of Daily Living Scale; SS-QOL-2, Stroke Specific Quality of Life Scale; PGIS, Patients' Global Impressions Scale; CGIS, Clinical Global Impressions Scale; ACT, auditory consonant trigrams; TMT, Trail Making Test; verb., verbal; LNS, letter number sequencing; PCL, Post-Traumatic Stress Disorder Checklist; acc., accuracy; RT, reaction time; TAP, Test of Attentional Performance; TEA, Test of Everyday Attention*.

The phasic alertness training task requires controlling the speed of a vehicle to avoid hitting obstacles. The vigilance training tasks include identifying damaged objects in a production line and identifying changes in airplane movements on a flight radar. The selective attention training tasks requires to respond quickly when previously defined objects appear on the screen and to ignore others. The divided attention training task requires to monitor three parameters (both visual and auditory) and press whenever either of these parameters fall outside a certain range (Sturm et al., 1997).

#### Specific vs. non-specific attention training

AixTent was used in two studies that examined whether attention training should be specifically aimed at the impaired domain or whether general attention training could also result in improvements of a specific attention domain. Participants received the training for one of at least two attention domains that were impaired. Thus, the affected target domain received specific training, whereas the other received a non-specific training. After adjusting for multiple testing, the training improved only (Sturm et al., 2003) or mostly (Sturm et al., 1997) the target domain. This does not imply that the training resulted only in near transfer, as the tasks used for the training differed from the outcome measures. Moreover, the vigilance training improved selective attention, and the basic alertness training improved more complex selective and divided attention (Sturm et al., 1997). Thus, some far transfer effects to other domains seemed to be present. The authors concluded that attention training should be specific. This may in particular be the case when cognitive functions are hierarchical, where more basic functions should be trained first followed by more complex cognitive functions.

#### Basic attention training

These results (Sturm et al., 1997, 2003) also suggest that improvements in basic cognitive functions may generalize toward improving more complex cognitive functions but not the other way around. This implication indeed seemed to hold (at least partially) in a single case study and in a small matched control study of a basic alertness training (Hauke et al., 2011; van Vleet et al., 2015). In the single case study, the training effect was largest for alertness, that is, for the attention domain being trained (Hauke et al., 2011). During the multiple baseline assessments there was no improvement of alertness, suggesting the effect was specific to the training period. Training this basic attention domain not only improved alertness, but also focused attention, vigilance, and divided attention (both visual and auditory). These improvements remained stable 6 months after training completion. The participant also reported subjective improvements of attention to a normal level. She reported lower levels of fatigue, but still not at a normal level.

All improvements were observed already within six or eight training sessions, and subsequently, performance remained stable, suggesting that a few sessions suffice to train attention. Alternatively, placebo effects may have been present as only three training sessions already had a significant effect on alertness. Moreover, the significant improvements in the attention domains not being trained were already observed during the baseline period. Thus, it is impossible to separate the effect of the basic attention training in these more complex attention domains.

Basic attention training also resulted in improvements of non-trained executive functioning in a small matched control study (van Vleet et al., 2015). Three mild TBI patients with complaints of executive functioning received 4.5 h of alertness training. Clinically significant improvements (z-score difference > 1) were found on the individual level. All three patients clinically improved on two or three of the five executive functioning tasks and on an attention task. Conversely, one of the two control participants improved on only one of the five executive functioning tasks. These two small studies did not provide *p*-values (Hauke et al., 2011) or did not perform statistical testing (van Vleet et al., 2015). Thus, evaluation of the effects after adjustment for multiple testing could not be performed.

#### Hierarchical attention training

The above findings suggest that training basic attention may result in improvement of more complex attention and executive functioning. The effect of a hierarchical approach to attention training was examined in four patients who suffered an acquired brain injury (Gauggel and Niemann, 1996). During the first week of the study alertness was trained, followed by vigilance training and selective attention training, and in the last week divided attention was trained.

Participants were studied within 3–16 months post onset, and two already showed improvements during the baseline phase. It was, therefore, impossible to conclude whether the improvement after training of these two participants on an attention task was due to the hierarchical training. The effect of training did not generalize to ratings of life satisfaction and depressive feelings, or to non-trained cognitive domains.

The inconclusive results of this small study are not in line with the previous studies. Since this study presented the training in a hierarchical manner, one would expect clear improvement in attention and maybe even in other cognitive domains. The training duration of 12.5 h may have been insufficient as multiple training tasks were used. No outcome measures related to executive functioning were included. Thus, it is impossible to determine whether a hierarchical approach results in improvements of executive functioning.

#### Training of multiple attention domains

Several other studies that also used tasks from multiple attention domains, but which did so for each training session in a non-hierarchical way, showed mixed results. Tasks used to train attention can be either basic or can be made more interesting by adding graphics and by integrating them into a game-like environment (such as AixTent). Zickefoose et al. (2013) compared both of these types of attention training within one study. Their sample consisted of four participants who had suffered a severe traumatic brain injury (TBI) at least 3 years ago. Within an A-B-A-C-A design, participants first started with 20 half-hour sessions of either the basic Attention Process Training-3 or several game-like attention tasks of the Lumosity website. Next, they followed 20 sessions of the other training.

Participants improved on the training tasks; they especially enjoyed Lumosity and were motivated to continue the training. Improvements were only observed in a subset of the non-trained tasks. One of the attention tasks appeared to suffer from a ceiling effect. One participant significantly improved after both training programs, whereas the other three participants showed both improvements and decrements in performance. Nevertheless, when there was an improvement, it was not only in basic attention but also in the more complex divided attention. The patterns of improvement revealed that generalization effects in this study, if any, were not very convincing. The authors suggested that the effects could be larger for less severely affected patients or for those receiving training early after injury. In addition, similar to Gauggel and Niemann (1996), the training occurred two times per week for 4 weeks, giving a total of 20 h, which may have been too short for generalization to occur.

In a RCT, Prokopenko et al. (2013) trained post-acute stroke patients with mild cognitive impairment and mild dementia. They based their training on several tasks used in neuropsychological assessments and kept the graphics of the training simple. Two weeks of training, focused on improving attention and visual and spatial abilities, apparently resulted in near transfer effects. After the training, participants in the intervention group (*n* = 24) scored significantly higher than the care-as-usual control group (*n* = 19) on tasks that closely resembled tasks used in the training.

Far transfer effects, however, were only observed in one out of seven tasks (a screening of executive functioning). Instrumental activities of daily living, mood, and quality of life did not improve (Prokopenko et al., 2013). None of the significant near and far transfer effects would survive adjustment for multiple testing. The measures that did not improve were very general and may have been insensitive to training effects. Furthermore, even though relatively long compared to other attention training programs, this training was still short. It only involved 15 h spread over 2 weeks and over training tasks of multiple attention domains, and the training tasks were not very attractive, which may have influenced participants' motivation. Nevertheless, only the intervention group reported subjective improvement of symptoms after the 2-week period, based on a rating of training satisfaction.

One study did not find any training effects. Ten patients who were within 9 months post severe head injury followed a speed of processing training that consisted of simple reaction time tasks, some of which involved the inhibition of responses (Ponsford and Kinsella, 1988). At a group level, the training did not add to the effect of spontaneous recovery. In half of the participants there only appeared to be a training effect when the therapist gave feedback about performance on the training tasks. This suggests that giving insight into the participant's performance, and thereby potentially increasing their motivation for the training, is important.

The training duration was 7.5 h in total, which is nearly half as long as the attention training programs we discussed so far. In addition, multiple tasks were used in the training, thus the training may not have been long enough to result in improvements. Another study that did show some effect of training with multiple tasks had at least 15 h of training (Prokopenko et al., 2013). In addition, the participants of Ponsford and Kinsella's study suffered very severe head injury, so that their brain damage may have been too severe for restitution training to be effective.

A strong point of the study by Ponsford and Kinsella is that they used an appropriate method to control for effects of spontaneous recovery. They did not only use a multiple baseline design, but they also investigated whether the *increase* in performance was larger during the training period than during the baseline period. The lack of training effect after correcting for spontaneous recovery underscores the necessity of adequate control groups or multiple baseline measurements.

#### Conclusion of attention training studies

Based on the results of these studies, it is still unclear what an attention training should consist of to be effective. Neither the Attention Process Training-3 nor Lumosity training proved to be superior to the other (Zickefoose et al., 2013). Participants preferred the graphically stimulating Lumosity training, compared with the basic training. This indicates the importance to adjust training environments to the preferences of the trainee. Graphics can make the training more interesting. However, our experience in clinical practice is that, for example, flashing graphics and sounds may be distracting for certain patients. This potential trade-off should be investigated further.

Training is most effective in the attention domain that is specifically trained (Sturm et al., 1997, 2003; Hauke et al., 2011; Prokopenko et al., 2013; van Vleet et al., 2015). Attention may be seen as a hierarchy, in which training of basic attention can improve more complex attention. It is not clear yet whether training complex before basic attention can result in overloaded basic attention and consequently in deteriorated performance, as was suggested by Sturm et al. (1997, 2003). It is also not clear whether a hierarchical training would be superior to a training that either focuses on one attention domain or that combines several attention domains per session (Gauggel and Niemann, 1996; Prokopenko et al., 2013).

Several types of attention training transferred to at least some executive function tasks (Sturm et al., 1997; Hauke et al., 2011; Prokopenko et al., 2013; Zickefoose et al., 2013; van Vleet et al., 2015), but not to an abstraction task (Ponsford and Kinsella, 1988). Ecologically valid measures were not often included (Sturm et al., 1997, 2003; Zickefoose et al., 2013) or were only very general (Gauggel and Niemann, 1996; van Vleet et al., 2015). Of these ecologically valid measures, objective attention (Ponsford and Kinsella, 1988), subjective IADL (Prokopenko et al., 2013), and life satisfaction (Gauggel and Niemann, 1996; Prokopenko et al., 2013) did not significantly improve. Only subjective attention improved (Hauke et al., 2011), whereas results for mood were inconclusive (Gauggel and Niemann, 1996; Prokopenko et al., 2013; van Vleet et al., 2015). Finally, it is important to provide feedback to the participant (Ponsford and Kinsella, 1988). Except for Sturm et al. (1997, 2003) and Prokopenko et al. (2013), studies did not correct for multiple testing and did not provide *p*-values. Thus, we were unable to take into account any distortions due to multiple statistical testing.

#### Limitations

The inter-individual differences in training outcomes may be due to factors such as lesion characteristics. None of the studies determined the extent of brain damage. One would expect that not everybody benefits equally from restitution-based training, assuming it depends on the residual functionality of the network being trained (Robertson and Murre, 1999). The study that included very severe head injury patients (Ponsford and Kinsella, 1988) did not reveal any transfer effects of the training, whereas the studies that included mild brain injury patients showed some transfer effects (e.g., Prokopenko et al., 2013; van Vleet et al., 2015). Future studies should, therefore, include imaging measures that can provide insight into the severity of damage to brain networks. Other limitations will be outlined in the discussion section.

### Combined working memory and attention training

Non-specific training may result in beneficial effects when the aim is not to train one specific domain. Most studies that combined several cognitive domains included attention and working memory games (see Table 4A for an overview of a combined training study with double baseline and Table 4B for studies with single baseline). A variety of programs were used. One program used by two studies was RehaCom.

**Table 4A:** **An overview of combined working memory and attention training study with double baseline measurement**.

**Study**	**Population**	**Sample size**	**Training (focus)**	**Duration training (actual M)**	**Setting**	**Outcome measures**	**Significance of comparisons of pre-training (t_0_), second baseline (t_0b_), post-training (t_1_), and follow-up (t_2_)**							**Multiple testing adjusted**	**Ecological valid measure**	**Train. improv. Evaluated**	**Reported conflicts of interest**
							**Within Ss**				**Between Ss**						
							**t_0b_–t_0_**	**t_1_–t_0_**	**t_2_–t_0_**	**t_2_–t_1_**	**t_1_–t_0_**	**t_2_–t_0_**	**t_2_–t_1_**				
Ruff et al., 1994	Severe head injury, post-acute—chronic (> 6 m post onset)	*N* = 15: no control	THINKable (attention, memory)	2 h/d, ??? w = 2 × 20 = 40 h (unkn.)	Rehab.	Pc Attention	.	±	×	×	×	×	×	No	Yes	No	Yes, research was supported by company that developed the training
						Pc Memory I	.	−	×	×	×	×	×				
						Pc Memory II	.	±	×	×	×	×	×				
						Pc Memory III	.	−	×	×	×	×	×				
						Ruff 2 & 7 SAT	.	−	×	×	×	×	×				
						Digit symbol	.	+	×	×	×	×	×				
						Cont. perf. I	.	−	×	×	×	×	×				
						Cont. perf. II	.	−	×	×	×	×	×				
						RAVLT - direct	.	±	×	×	×	×	×				
						RAVLT - delayed	.	−	×	×	×	×	×				
						Corsi - direct	.	±	×	×	×	×	×				
						Corsi - delayed	.	−	×	×	×	×	×				
						WMS:											
						Information	.	−	×	×	×	×	×				
						Orientation	.	−	×	×	×	×	×				
						Mental control	.	±	×	×	×	×	×				
						Logical memory	.	±	×	×	×	×	×				
						Digits forward & backward	.	−	×	×	×	×	×				
						Visual reprod.	.	−	×	×	×	×	×				
						Assoc. learning	.	−	×	×	×	×	×				
						Subj. att. - self	.	−	×	×	×	×	×				
						Subj. att. - proxy	.	±	×	×	×	×	×				
						Subj. mem. - self	.	±	×	×	×	×	×				
						Subj. mem.-proxy	.	+	×	×	×	×	×				
						Beck depr.	.	−	×	×	×	×	×				

*+, significant and survives adjustment for multiple testing (Bonferroni-Holm adjustment is based on the number of outcome measures used at that time-point); ±, significant but doesn't survive adjustment; -, not significant; ×, not included in analysis;., not mentioned in methods*.

*M, mean; Train. improv., training improvements; Ss, subjects; N, sample size; m, months; w, weeks; h/d, hours per day; h, hours; unkn., unknown; rehab., rehabilitation center*.

*Abbreviations neuropsychological tests: Pc, personal computer; SAT, Selective Attention Test; Cont. perf., Continuous performance; RAVLT, Rey Auditory Verbal Learning Test; WMS, Wechsler Memory Scale; reprod., reproduction; Assoc., Associated; Subj., subjective; att., attention; mem., memory; depr., depression*.

**Table 4B:** **An overview of combined working memory and attention training studies with single baseline measurement**.

**Study**	**Population**	**Sample size**	**Training (focus)**	**Duration training (actual M)**	**Setting**	**Outcome measures**	**Significance of comparisons of pre-training (t_0_), post-training (t_1_), and follow-up (t_2_)**							**Multiple testing adjusted**	**Ecological valid measure**	**Train. improv. Evaluated**	**Reported conflicts of interest**
							**Within Ss**				**Between Ss**						
							**t_1_–t_0_**		**t_2_–t_0_**	**t_2_–t_1_**	**t_1_–t_0_**	**t_2_–t_0_**	**t_2_–t_1_**				
							**CG**	**IG**									
Chen et al., 1997	CHI, post-acute—chronic (*M* = 15.9 m)	*N* = 40: IG = 20 CAU = 20	Bracy Process Approach (att., visual- spatial orientation, memory, problem-solving)	Differed per participant (unkn.)	Rehab.	Verbal IQ	+	+	.	.	×	.	.	Yes, *p* < .004 with Bonfer-roni correct.	No	No	Not reported
						Performance IQ	+	+	.	.	×	.	.				
						Full scale IQ	+	+	.	.	×	.	.				
						WAIS-R:											
						Information	(+)/-	+	.	.	×	.	.				
						Vocabulary	−	+	.	.	×	.	.				
						Digit span	(+)/-	+	.	.	×	.	.				
						Arithmetic	−	−	.	.	×	.	.				
						Comprehension	−	−	.	.	−	.	.				
						Similarities	(+)/-	+	.	.	×	.	.				
						Picture compl.	+	+	.	.	×	.	.				
						Picture arrange.	+	+	.	.	×	.	.				
						Block design	+	+	.	.	−	.	.				
						Object assem.	+	+	.	.	−	.	.				
						Digit symbol	+	+	.	.	×	.	.				
						Category test	(+)/-	+	.	.	−	.	.				
						TMT-A	+	+	.	.	−	.	.				
						TMT-B	+	+	.	.	−	.	.				
						WCST	(+)/-	+	.	.	−	.	.				
						Mental control	−	−	.	.	−	.	.				
						Digit span forw.	−	(+)/-	.	.	−	.	.				
						Digit span rev.	(+)/-	+	.	.	−	.	.				
						Logical mem.	−	+	.	.	−	.	.				
						Logical mem. DR	(+)/-	+	.	.	−	.	.				
						Visual reprod.	−	+	.	.	−	.	.				
						Visual reprod DR	(+)/-	−	.	.	−	.	.				
						Paired assoc.	×	×	.	.	×	.	.				
						Paired assoc. DR	×	×	.	.	×	.	.				
De Luca et al., 2014	Severe brain injury, post-acute phase (3–6 m post onset)	*N* = 34: IG + CAU = 15 CAU = 19	Based on several web-based programs (memory, EF, abilities of thinking)	??? min, 3 d/w, 8 w (unkn.)	Rehab.?	MMSE	−	+	×	.	+	×	.	No	Yes, question-naires but not specific about EF	No	Not reported
						LCF	±	+	×	.	+	×	.				
						RML	−	+	×	.	+	×	.				
						RAVLT - direct	−	+	×	.	+	×	.				
						RAVLT - delayed	−	+	×	.	+	×	.				
						Att. Matrices	−	+	×	.	+	×	.				
						CV Fluency	−	+	×	.	±	×	.				
						LV Fluency	−	+	×	.	+	×	.				
						Constr. Apraxia	±	±	×	.	+	×	.				
						Barthel Index	+	+	×	.	−	×	.				
						ADL	±	+	×	.	±	×	.				
						IADL	±	+	×	.	±	×	.				
						HRS anx.	−	−	×	.	±	×	.				
						HRS depr.	−	+	×	.	±	×	.				
Fernandez et al., 2012	ABI (Stroke or TBI), mostly chronic (1-5 y post onset)	*N* = 50 no control	RehaCom software package (divided att., concentration, RT, audio and visual memory)	50 min, 5 d/w, 12 w = 50 h (unkn.)	Lab, 4 at a time with presence two specialists	MMSE	.	−	.	.	.	.	.	No	No	No	Not reported
						WMS:											
						Information	.	^*^	.	.	.	.	.				
						Orientation	.	^*^	.	.	.	.	.				
						Mental control	.	^*^	.	.	.	.	.				
						Logical memory	.	^*^	.	.	.	.	.				
						Digits forward and backward	.	^*^	.	.	.	.	.				
						Visual reprod.	.	^*^	.	.	.	.	.				
						Assoc. learning	.	^*^	.	.	.	.	.				
						Mem. quotient	.	^*^	.	.	.	.	.				
						TMT-A	.	^*^	.	.	.	.	.				
						TMT-B	.	−	.	.	.	.	.				
Gray et al., 1992	mild-severe ABI, post-acute—chronic (7 w–10 y) with attention deficits	*N* = 31: IG = 17 aCG = 14 (recrea-tional computing without time pressure)	Unkn. (att. involved in processes of control)	14 times 75 min, 2 d/w = 17.5 h (15.35h)	Rehab.?	Digit span forw.	×	×	×	×	−	−	.	No	No, only a psycho-logical well-being measure but not specific about EF	No	Not reported
						Digit span rev.	×	×	×	×	−	±	.				
						PASAT:											
						Total	−	±	×	±	−	+	.				
						Longest string	−	−	±	±	−	±	.				
						Info proc. rate	−	±	×	+	±	±	.				
						Arithmetic	−	±	×	×	−	±	.				
						WCST error	×	×	×	×	−	−	.				
						WCST pers. error	×	×	×	×	−	−	.				
						Finger tapping	×	×	×	×	−	−	.				
						Word fluency	×	×	×	×	−	−	.				
						Letter cancel.:											
						Time	×	×	×	×	×	×	.				
						Error	×	×	×	×	−	−	.				
						Picture compl.	×	×	×	×	±	−	.				
						Time estimation	×	×	×	×	−	−	.				
						Logical mem.	×	×	×	×	−	−	.				
						Logical mem. DR	×	×	×	×	−	−	.				
						Rey - copy	×	×	×	×	−	−	.				
						Rey - delayed	×	×	×	×	−	−	.				
						Neale accuracy	×	×	×	×	×	×	.				
						Comprehension	×	×	×	×	−	−	.				
						Block design	×	×	×	×	−	±	.				
						Similarities	×	×	×	×	−	−	.				
						GHQ-28	−	−	−	−	−	−	.				
Lin et al., 2014	Stroke, post-acute (6–10 m) with EF and memory deficit	*N* = 34: IG = 16 pCG = 18	RehaCom software package (EF, memory)	1 h, 6 d/w, 10 w = 60 h (unkn.)	Rehab.?	WMS:								Not for NPA MRI *p* was set to < 0.005	No	No	None
						Information	−	−	.	.	×	.	.				
						Orientation	−	−	.	.	×	.	.				
						Mental control	−	+	.	.	×	.	.				
						Logical memory	−	+	.	.	×	.	.				
						Digits forward and backward	−	±	.	.	×	.	.				
						Visual reprod.	−	+	.	.	×	.	.				
						Assoc. learning	−	+	.	.	×	.	.				
						Memory quotient	−	+	.	.	×	.	.				
						TMT-A	−	+	.	.	×	.	.				
						TMT-B	−	−	.	.	×	.	.				
						Corr. between MRI & NPA	−	+	.	.	.	.	.				
						MRI	+	+	.	.	.	.	.				
Spikman et al., 2010	ABI, post-acute—chronic (3 m–39 y post onset) with dysexecutive complaints	*N* = 75: PC = 37 strategy training = 38	Cogpack (reaction speed, att., memory, planning)	20–24 times 1 h, 2 d/w, in 3 m = 22 h (unkn.)	Rehab.	Role Resum. List	+	+	+	−	- -	- -	- -	Yes, Bonfer-roni Holm correct.	Yes	No	Not reported
						DEX patient	+	+	+	×	−	−	.			
						DEX proxy	+	+	−^*^	×	−	−	.			
						DEX therapist	+	+	+	×	- -	−	.				
						Ex. Obser. Scale	+	+	+	×	- -	- -	.				
						QOLIBRI satisfac	+	+	−^*^	×	−	−	.		
						QOLIBRI burden	+	+	−^*^	×	−	−	.		
						BADS	+	+	+	×	−	−	.				
						TMT-B/A	−^*^	−^*^	−^*^	×	−	−	.		
						Stroop 3/2	−^*^	−^*^	+	×	−	−	.		
						ToL	+	+	−^*^	×	−	−	.				
						RAVLT - direct	+	+	×	×	−	×	.			
						RAVLT - delayed	−^*^	−^*^	×	×	−	×	.				
						Tx Satisf. Scale	.	.	.	.	.	.	−				
						Tx Goal Attain.	.	.	.	.	- -^a^	.	- -				
						Ex. Secret. Task	.	.	.	.	.	.	−				

+, significant (sign.) and survives adjustment for multiple testing (Bonferroni-Holm adjustment is based on the number of outcome measures used at that time-point); ±, sign. but doesn't survive adjustment; -, not sign.; (+)/-, not sign. but would be without adjusting; ^*^, sign. but unknown whether it would survive adjustment; −^*^, not sign. but unknown whether it would be sign. without adjustment; - -, CG is sign better than IG; ×, not included in analysis;., not mentioned in methods; a, not measured at baseline.

*M, mean; Train. improv., training improvements; Ss, subjects; IG, intervention group; CG, control group; aCG, active control group; pCG passive control group; CAU, care as usual; strat., strategy group; PC, personal computer; N, sample size; y, years; m, months; w, weeks; d/w, days per week; h, hours; min, minutes; CHI, closed head injury; ABI, acquired brain injury; TBI, traumatic brain injury; EF, executive functioning; att., attention; RT, reaction time; unkn., unknown; rehab., rehabilitation center; NPA, neuropsychological tests; correct., correction*.

*Abbreviations neuropsychological tests: WAIS-R, Wechsler Adult Intelligence Scale revised; compl., completion; arrange., arrangement; assem., assembly; TMT, Trail Making Test; WCST, Wisconsin Card Sorting Test; forw., forward; rev., reversed; mem., memory; DR, delayed recall; reprod., reproduction; assoc., associated; MMSE, Mini-Mental State Examination; LCF, Levels of Cognitive Functioning; RML, Reversal Motor Learning; RAVLT, Rey Auditory Verbal Learning Test; Att. Matrices, Attentive Matrices; CV, Category Verbal; LV, Letter Verbal; Constr., Constructional; (I)ADL, (Instrumental) Activities of Daily Living Scale; HRS, Hamilton Rating Scale; anx., anxiety; depr., depression; WMS, Wechsler Memory Scale; PASAT, Paced Auditory Serial Attention Test; proc., processing; pers., perseverative; cancel., cancelation; GHQ-28, General Health Questionnaire; corr., correlation; Role Resum. List, Role Resumption List; DEX, Dysexecutive Questionnaire; Ex. Obser. Scale, Executive Observation Scale; QOLIBRI, Quality of Life after Brain Injury; satisfac, satisfaction; BADS, Behavioural Assessment of the Dysexecutive Syndrome; ToL, Tower of London; Tx Satisf. Scale, Treatment Satisfaction Scale; Tx Goal Attain., Treatment Goal Attainment; Ex. Secret. Task, Executive Secretarial Task*.

#### RehaCom

The RehaCom training consists of several graphical games that adapt to the performance of the participant and use a variety of stimuli such as playing cards. The training focusses on several cognitive domains. First, selective attention tasks where, for example a particular image needs to be found amongst several distracter images. Second, working memory tasks included to click on the playing cards that were shown before; at higher levels the cards need to be reproduced in reversed order. Finally, executive function was trained via divided attention tasks such as control the speed of a car while listening to the radio; or buying items from a shopping list while the purchases must fit within a certain budget.

#### Non-specific training

The two studies that evaluated RehaCom found generalizing effects to nearly all tasks used. Training improved performance on seven working memory tasks (both auditory and visual) and an attention task (Fernandez et al., 2012; Lin et al., 2014). Even though the authors did not adjust for multiple testing, the effect found by Lin et al. (2014) would remain significant if adjusted. No improvements were observed in the control group (*n* = 18), which received no training (Lin et al., 2014). However, the two groups were not directly compared, and Fernandez and colleagues did not include a control group. Thus, the results may be due to factors other than the training. Although these two studies used training programs of 50–60 h that included executive function tasks, there were no significant improvements on a task that is frequently used to measure executive function (i.e., the Trail Making Task version B). Both studies only used one outcome measure to assess executive function, which may have been insufficient to capture the spectrum of executive functioning.

A RCT using a similar, non-specific, 8-week training did reveal significant improvements on two tasks measuring executive function (De Luca et al., 2014). Participants who completed this training (*n* = 15) improved on 13 of the 14 outcome measures. This included objective neuropsychological measures of executive functioning, attention, and memory. It also included subjective functional and behavioral scales for daily living (De Luca et al., 2014). These improvements, except for one scale measuring functional performance in everyday life, were significantly larger than in a control group (*n* = 19), which received care as usual. Even though the authors did not adjust for multiple testing, 12 outcome measures (including the executive function measure) would survive adjustment for multiple testing. This suggests that the training resulted in improvements that generalized to untrained tasks.

As the study sample consisted of post-acute patients who had suffered severe brain injury, these positive results do not agree with the studies discussed earlier that failed to reveal (conclusive) transfer of training effects after severe brain injury (Ponsford and Kinsella, 1988; Zickefoose et al., 2013). De Luca and colleagues did not provide detailed information about the training or session duration. It is, therefore, impossible to evaluate which elements of the training resulted in these positive effects. For the subjective outcome measures it should be kept in mind that the control group received less attention, whereas the intervention group received 24 extra sessions, which may have contributed to a larger placebo effect.

Spikman et al. (2010) evaluated a 20-h Cogpack training (*n* = 37) and compared it to a multifaceted strategy training (*n* = 38). They found improvements in objective and subjective executive functioning in both groups. A far transfer effect was also observed in short-term memory and in subjective quality of life. All but the subjective quality of life improvements remained significant 6 months after training completion. These results were adjusted for multiple testing, which suggests that effects were likely to be true effects. Nevertheless, the Cogpack computer-training group never improved significantly more than the comparison group. Conversely, immediately after training, the strategy group improved more than the computer-training group on two executive function scales. These were, however, both rated by the therapist who was not blind to treatment condition. Neither of the training programs showed improvements in two tasks commonly used to measure inhibition or executive functioning. This was similar to what was found by Fernandez et al. (2012) and Lin et al. (2014). These two tasks may have been less vulnerable to retest effects than the other two executive function tasks that did show improvements after the training.

Both groups were equally satisfied with training, reported less executive dysfunction 6 months after the training, and felt that they started to participate again in social and vocational life. There was no evidence that the Cogpack computer training resulted in better outcome compared to strategy training. However, since improvements were observed in both groups a waiting list control group would be necessary to confirm whether the effects were specific to the training. Nevertheless, even if the improvements were mere retest effects, they may have had a positive effect on the participants' mood and motivation to continue a rehabilitation program.

In sum, training that combines memory and attention tasks resulted in transfer to working memory and attention tasks that were not trained. The extent of these training effects on executive function remains unclear as most studies included only one executive function task (Fernandez et al., 2012; Lin et al., 2014). The studies that did include multiple executive function tasks, did find improvements on most of these tasks (Spikman et al., 2010; De Luca et al., 2014), but the results of Spikman and colleagues were also found in their comparison group.

#### More specific training

Two studies used training programs that were primarily focused on one cognitive domain. The main focus of the training used in the RCT by Gray et al. (1992) was attention; we report this training in this section as it also included set shifting. The training consisted of approximately 14 sessions of 1–1.5 h, resulting in about 15 h of training (*n* = 17). The active control group (*n* = 14) could play computer games of their choice that did not involve time pressure, and they trained 12.7 h on average.

Twenty-one outcome measures were used, but only two significant group differences were found. Moreover, these effects disappeared when time since onset of brain injury and premorbid IQ were taken into account. Thus, the authors failed to find any far transfer effects immediately after training. However, 6 months after training completion, the experimental group did show a significant improvement compared to the control group on several tasks that were similar to the focus of the training. This effect remained after controlling for premorbid IQ and time since onset. The authors suggested that these improvements were already visible immediately after training but only reached significance at follow-up. They concluded that the training only had an effect on targeted functions but failed to generalize to cognitive functions that were not trained. This study stresses the importance of follow-up measurements.

Although the training included several executive functioning tasks, the experimental group did not improve significantly more on these tasks than the control group. Both groups showed large variability in baseline scores on the executive functioning task similar to the training. Perhaps the study lacked sufficient statistical power to reveal a significant improvement. Furthermore, as the control group could freely choose the computer tasks, it was unclear which they performed and whether these tasks improved cognition.

Another study that used a specific training consisted of either memory tasks or attention tasks (Ruff et al., 1994). These two training programs were compared in a multiple baseline design with 15 participants who had suffered severe head injury. However, both groups were pooled for statistical analyses, so that unfortunately training specific effects could not be identified. Results revealed that a proxy, who knew that their acquaintance followed the training, rated significant improvements in both attention and memory. Participants themselves rated that they significantly improved in memory, but not in attention. The training also improved objective short-term memory performance but failed to influence long-term memory. Depression scores did not consistently change after the training.

The authors did not include a control group, nor did they adjust for multiple testing. Only the effect on a processing speed task and the proxy ratings on memory would remain significant if they would have been adjusted. As the training tasks were not described, it is impossible to evaluate the results in light of the training. Moreover, the absence of executive function outcome measures makes it impossible to conclude whether the effects generalized to executive functions.

#### Hierarchical training

In a retrospective study a hierarchical computer training was evaluated in closed head injury patients (Chen et al., 1997). The training started with basic cognitive functions and subsequently focused on more complex functions. Due to the retrospective nature of this study, training duration and interval between training and follow-up differed between participants. No differences were found between the care-as-usual group (*n* = 20) and the computer-training group (*n* = 20) in four composite scores of the cognitive domains on which the training focused. Nevertheless, when evaluating each task separately, the computer-training group gained significantly on 20 tasks compared with a mere 10 tasks in the care-as-usual group after adjusting for multiple testing (see Table 4B for measures that would be significant without adjustment). This included an executive function task, an attention task, and some memory tasks. Participants were not randomly assigned to groups, and the groups differed significantly in time since onset and length of treatment. Even though these two variables were added as covariates, still other factors may have influenced the treatment effects.

#### Conclusions and limitations of combined working memory and attention training

Training programs combining attention, working memory, and other executive function tasks did not show consistent objective executive functioning improvements. This may be due to the small number of tasks used in some studies to measure executive functioning, to the large variability of baseline scores on these tasks, and to the often small sample sizes and ensuing low statistical power of these studies.

Subjective executive function improvements were noted by the participants themselves and by their proxies and therapists (Spikman et al., 2010). Other subjective improvements were reported for attention and memory (Ruff et al., 1994), everyday life functioning (De Luca et al., 2014), and quality of life (Spikman et al., 2010). Effects on mood were inconclusive; whereas reductions in anxiety were found, psychological well-being did not improve (Gray et al., 1992) and depression levels were only reduced in one of two studies in which it was measured (De Luca et al., 2014). Except for depression, these subjective ratings were never measured in more than one study. Thus, replication is clearly needed. Moreover, studies that included an active control group found improvements in both groups (Spikman et al., 2010) and did not find any group differences (Gray et al., 1992; Spikman et al., 2010). The other studies either included a passive control group or no control group at all, and thus results could be due to placebo effects.

Both objective auditory and visual memory commonly improved (Chen et al., 1997; Fernandez et al., 2012; Lin et al., 2014), but this was the case for immediate recall and rarely for delayed recall (Ruff et al., 1994; Spikman et al., 2010; De Luca et al., 2014). Similarly, objective attention also improved (Gray et al., 1992; Ruff et al., 1994; Chen et al., 1997; Fernandez et al., 2012; De Luca et al., 2014; Lin et al., 2014). Some of these effects were revealed only at the long term (Gray et al., 1992) and some effects were not significantly larger compared with the control group (Chen et al., 1997; Spikman et al., 2010). Most training programs did include a memory or attention component, and therefore, improvements in these domains were expected.

Improvements in non-trained objective outcomes were also frequently reported. General cognition improved more than in the control group (De Luca et al., 2014). Furthermore, increased participation in everyday life (Spikman et al., 2010), processing speed (Ruff et al., 1994; Chen et al., 1997), IQ, and problem solving (Chen et al., 1997) were found. Conversely, improvements of verbal reasoning were inconsistent (Gray et al., 1992; Chen et al., 1997). The within group effects were not compared with a control group (Ruff et al., 1994; Chen et al., 1997) or the effects were also found in the control group (Spikman et al., 2010). Thus, even though these results seem promising, they need to be interpreted cautiously because of the lack of proper control groups, and they need to be replicated with improved methodological designs.

In contrast to attention specific training (Ponsford and Kinsella, 1988; Zickefoose et al., 2013), the training programs including multiple cognitive domains were effective after severe brain injury (Ruff et al., 1994; De Luca et al., 2014). Training also appeared to be effective for both post-acute patients (De Luca et al., 2014; Lin et al., 2014) and for those who were in the chronic phase (Fernandez et al., 2012). Finally, stroke patients (Lin et al., 2014) as well as patients with other etiologies (Ruff et al., 1994; Chen et al., 1997; Spikman et al., 2010; Fernandez et al., 2012; De Luca et al., 2014) seemed to benefit from the training.

### Neural effects of computer training

Nordvik et al. (2014) emphasized that most computer-based training studies do not investigate the effects on a neural level. In their overview, they summarize evidence for both gray and white matter changes after training certain skills in the healthy population (Nordvik et al., 2014). Within the stroke population, imaging is rarely used as an outcome measure. However, recently two studies reported both functional and structural changes in the brain after restitution-based training. One of these studies included strategy education as part of their training (Nordvik et al., 2012). Even though this study, therefore, does not fulfill our inclusion criteria, we still report it here, because the main elements of the training were two types of computer training, and because such imaging studies are sparse.

In a single case study, both a general computer training (focusing on five cognitive domains) and the specific Cogmed working memory training, were combined with a weekly session which included discussions about possible strategy use. Structural white matter connectivity measures changed during the training period and were stable when the participant was not training (Nordvik et al., 2012). Visual inspection of the data revealed that both training programs improved working memory. The connectivity measure correlated with working memory.

Functional connectivity also changed after the training used by Lin et al. (2014). As mentioned before, both working memory and attention improved after this training. This improvement was related to increased functional connectivity of several brain areas. The control group did not show any improvements in working memory, attention, or executive function after the training. The regional functional connectivity of this group did, however, significantly decrease after the period without training, but these changes did not correlate with cognitive performance. Although changes in functional connectivity were observed in both groups, this suggests that these changes were only related to the training effects in the intervention group and not in the control group.

It is important to note that brain changes can occur even when no behavioral changes are measurable. As both increased and decreased activity can be interpreted positively (i.e., increased communication vs. more parsimonious and efficient communication, respectively), one should preferably have a clear a-priori hypothesis and include healthy aged matched controls. Using non-invasive brain imaging is still relatively new in the field of brain training, which will be able to provide more insight into its effectiveness.

## Discussion

### Summary of results

In this review we aimed to determine whether computer-based restitution training can improve executive functions. Two of the studies we reviewed were of high quality because they were RCTs with active control groups and a sufficiently large sample size (Gray et al., 1992; Spikman et al., 2010). The intervention training groups in these studies did not improve more than the active control groups.

All other studies suffered from important methodological limitations. Consequently, their more positive results should be interpreted with caution. Results from the RCTs that included passive control groups, thus not correcting for potential placebo effects, revealed that training resulted in near transfer effects (Westerberg et al., 2007; Lundqvist et al., 2010; Akerlund et al., 2013; Prokopenko et al., 2013). Far transfer effects were also found, but mostly in tasks that were somehow related to the trained cognitive function (Westerberg et al., 2007; Lundqvist et al., 2010; Akerlund et al., 2013; Prokopenko et al., 2013; De Luca et al., 2014; Lin et al., 2014). Subjective improvements were not conclusively demonstrated but transfer was observed in several studies (Westerberg et al., 2007; Lundqvist et al., 2010; Björkdahl et al., 2013; De Luca et al., 2014). Spikman et al. (2010) found similar results within their intervention group (thus without comparing it to the active control group).

Effects on executive function remain inconclusive. Four studies found no improvements (Ponsford and Kinsella, 1988; Fernandez et al., 2012; Akerlund et al., 2013; Lin et al., 2014), five found improvements in part of the measures (Gray et al., 1992; Chen et al., 1997; Westerberg et al., 2007; Spikman et al., 2010; Zickefoose et al., 2013), and seven found improvements in all of their executive function outcome measures (Sturm et al., 1997, 2003; Lundqvist et al., 2010; Hauke et al., 2011; Prokopenko et al., 2013; De Luca et al., 2014; van Vleet et al., 2015). These effects were usually based on only one or two tasks. One particular working memory and attention measure (i.e., Paced Auditory Serial Addition Test; PASAT (Gronwall, 1977)) showed training effects in all three studies that included this task as an outcome measure (Gray et al., 1992; Westerberg et al., 2007; Lundqvist et al., 2010). This concerned studies of working memory training and studies of combined working memory and attention training. The PASAT seems to be a sensitive task to training effects and is suitable to be included in future studies. Three studies did not evaluate training effects on executive functioning (Ruff et al., 1994; Gauggel and Niemann, 1996; Björkdahl et al., 2013).

Six studies evaluated long-term outcome (Ponsford and Kinsella, 1988; Gray et al., 1992; Lundqvist et al., 2010; Spikman et al., 2010; Akerlund et al., 2013; Björkdahl et al., 2013). Transfer effects mostly remained stable several months after training. In the RCT with an active control group of Gray and colleagues, the only significant effects were observed at long-term follow-up. Only two studies evaluated the neural effects of training (Nordvik et al., 2012; Lin et al., 2014). They found that both structural and functional changes were related to training improvement.

### What are the effective elements of training, and who benefits?

It remains unclear which patients benefit from training and which training elements are essential. Positive results were observed in both severe and mildly affected patients in both the post-acute or the chronic phase. One study did not find any effects in a very severely affected post-acute sample (Ponsford and Kinsella, 1988). Both specific and general training programs seemed to be effective. Nevertheless, improvements were largest in the domain of the training itself, and results suggest that the function being trained should at least partially be targeted on the task where transfer is desired. The two hierarchical training programs failed to be effective, perhaps due to their methodological limitations. Training can be either basic or provided in a game-like environment. Participants showed a slight preference for the game-like training, not surprisingly, so training should be adjusted to the personal preferences of the patient. Finally, it is important to provide feedback.

### Limitations of the reviewed studies

#### Lack of control groups and blinding

The lack of proper control groups is one of the most important limitations of the studies reviewed here. Including a proper control group is important because spontaneous recovery can occur, and retest effects are common, especially for executive functioning tasks. A meta-analysis of attention training (not necessarily by computer) revealed that effect sizes of studies without control groups were always larger than effect sizes of studies with control groups (Park and Ingles, 2001). Similarly, transfer effects were absent in the current review when compared to an active control group (Gray et al., 1992; Spikman et al., 2010). Without proper controls it is impossible to draw conclusions about the nature of any effects. A passive control group will only correct for retest effects and spontaneous recovery, but not for placebo effects. An active control group controls for both placebo effects and Hawthorne effects (i.e., effects of being involved in something new and receiving attention). Nonetheless, the training interventions of the two active control groups used by Gray and Spikman were both potentially effective themselves, suggesting that both the experimental and the control training resulted in transfer effects. On the basis of our review we recommend that both an active control group and a passive control group should be included.

Placebo effects, for that matter, are not necessarily an objectionable phenomenon. Even if just being involved in something new results in placebo effects, it may improve the patients' quality of life, and motivate them for other types of rehabilitation. Long-term evaluation, which is currently lacking in most studies, is necessary to determine whether short-term training or placebo effects indeed benefit the patient.

Some may consider the use of control groups as controversial from an ethical point of view, because a potentially beneficial training is withheld from patients. Alternatively, multiple baseline measures, especially if baseline duration varies between participants, could filter out some of the effects of spontaneous recovery and retesting (as done by Ponsford and Kinsella, 1988). Also, the methodology of single-subject designs has improved considerably over the last decade, and it deserves to be applied more often (Dugard et al., 2011).

Blinding of both assessor and participant is another important factor for reliable assessment of outcomes. Only three studies blinded the assessors, and none of the studies reported that the participants were blinded. Blinding of the participants is of course difficult, but can be achieved when mock training is included. This is challenging, because the line between an effective training and a convincing control training is very thin.

#### Incomplete training descriptions

Most studies did not report the mean training time. In studies that did report the actual training time, this often differed from the training time as previously planned by protocol (e.g., Gray et al., 1992). Training duration and frequency are important in order to conclude whether behavioral improvements may be ascribed to the training, and whether neural changes may be likely. The median planned training duration of the reviewed studies was 15.6 h. This seems rather brief to obtain stable behavioral changes. The number of repetitions achieved within this time frame may also be insufficient for neural changes to occur (Kimberley et al., 2010).

The setting of the training was hardly ever described. In healthy elderly, training effects were smaller when training was done at home than when it was done in a group setting on site (Lampit et al., 2014). Face to face instructions also resulted in longer training sessions (Cruz et al., 2014) and in larger improvements (Man et al., 2006), than when they were given online and training was done at home. These factors could not be evaluated in the current review. The lack of description of the specific outcome parameters used, of relating the outcomes to training performance, of reports on conflicts of interest, and of evaluation of possible harmful effects of the training, all complicate evaluation of training effects. Without a clear description of all training tasks, it is impossible to determine whether an effect is evidence for far or near transfer.

#### Statistical considerations

Only four studies adjusted for multiple statistical testing. Currently, there is no consensus whether this correction is necessary for pre-planned analyses (Rothman, 1990; Curran-Everett, 2000; Glickman et al., 2014). Confirmatory studies need to correct for multiple tests that concern the same research question; exploratory studies are not required to do so (Bender and Lange, 2001). In any case, it seems advisable to report unadjusted *p*-values and confidence intervals, and interpret the results in light of the number of statistical tests performed, especially when many tests are done. Replication studies are needed with the same outcome measures that previously have shown transfer effects, to allow drawing firm conclusions. The reviewed studies hardly ever used the same training program or outcome measures, and thus replication is still lacking.

The sample sizes used in the studies were small. Only three studies had more than 20 participants per group, one of which did not include a control group. None of the studies reported an a-priori sample size calculation to determine the sample size needed to reveal clinically significant effects. It is likely that effect sizes in this research field are small or moderate at best (e.g., Corbett et al., 2015). Thus, the studies reviewed here may have been underpowered, in which case, however, one might ask whether such small effects are still clinically relevant. For better insight into the clinical relevance of training interventions, future studies should report effect sizes.

#### Outcome measures

Executive functioning was usually measured with only one task. As this is a very broad concept, a single task may not be enough to capture potential effects on executive functioning. The large variation of baseline performance on executive function tasks may mask potential individual improvements, which also remain undetected with small sample sizes.

Ecologically valid measures were rarely used. If used, they mostly consisted of subjective ratings and questionnaires. Ecologically valid measures are needed to evaluate real life benefits. Imaging was used in only two studies, and it was thus rarely possible to assess the training effects at the neural level. Results from imaging were promising, supporting the inclusion of imaging as an outcome measure in future studies.

#### Selection bias

Another issue is possible selection bias. Most likely, patients only participated if they had at least some affinity with computers. Patients were recruited via rehabilitation centers, and sometimes from only one center. The latter may reduce the generalizability of the results. The exclusion rate was not often reported, but in the Akerlund study it was very high (e.g., >50%) which again reduces generalizability.

### Limitations of this review

There are several limitations to this review, most of which are inherent to the novelty of the field. First, due to heterogeneity of outcome measures it was not possible to perform a meta-analysis. Second, we could not assess the risk of bias. It is possible, and maybe even likely, that publication biases exist in this field of research. Selection bias in the studies was also not assessed. The acquired brain injury population is very heterogeneous with many different outcome and impairment patterns. Studies used strict inclusion criteria, which reduces generalizability. Third, we excluded virtual reality studies. Virtual reality often involves the use of the whole body, which makes is difficult to distil whether the effect is due to cognitive retraining or to the physical exercise involved. A recent systematic review of virtual reality studies concluded that it can be effective in improving cognition (Larson et al., 2014). With virtual reality it is possible to safely recreate real life situations. This may, therefore, be a good future way for repeated practice of certain tasks requiring executive functions.

### Strength of this review

Computers are now widely available and there is a trend to do brain training in many patient populations. It is important to establish whether the effectiveness of restitution-based computer programs can be confirmed. Our review added to the results of the previously performed systematic review (Poulin et al., 2012) because we systematically evaluated 20 studies that provided restitution-based training. Results of our review can be used to improve the methodology of future studies.

## Conclusion

Most studies we reviewed suffered from methodological limitations. Samples were mostly small, appropriate control groups were often absent, and adjustment for multiple testing was rarely done. Consequently, it is difficult to draw firm conclusions about the effectiveness of training. With the current study designs, the effects reported may be due—at least in part—to spontaneous recovery, retest effects, or placebo effects.

Effects were most often reported on non-trained tasks that measured the function being trained. There were also reports of far transfer to non-trained tasks, but these tasks still mostly included some part of the function being trained. Training often increased subjective functioning, which is probably very important to motivate patients to continue following rehabilitation and to work on improvements. Overall, the results of these studies warrant continuation of research to determine whether restorative training methods can improve cognitive functioning. Computer training can easily be done at home, which is a cost effective way of improving motivation and subjective functioning, and hopefully of objective functioning after acquired brain injury.

The most important methodological improvements for future studies are that these should have larger sample sizes, both a stimulating but non-effective active control group and a passive control condition. Training periods should be longer and more stimulating training tasks adjusted to the preference and the ability level of the trainee should be used. Studies should also evaluate predictors of training outcome such as time since injury and symptom severity. Multiple outcome measures per cognitive domain without ceiling effects and with satisfactory ecological validity should be used. Long-term effects need to be evaluated and results should be replicated. The interpretation of the results should be in light of training progression and after appropriate adjustment for multiple testing. Effect sizes should be reported in order to evaluate clinical significance of results.

In this field it is a challenge to conduct well designed and sufficiently powered studies due to low budgets available, limited number of available patients, heterogeneity of the population, and ethical considerations. With this in mind, the currently reviewed studies provide valuable insights and emphasize the need of carefully designed RCTs for the future.

## Author contributions

Conception and design of the work: RV, JM, BS. Data acquisition: RV. Data analysis: RV, BS. Interpretation of data: RV, JM, DV, BS. Drafting and revising the work: RV, JM, DV, BS. Final approval of the version to be published: RV, JM, DV, BS. Agreement to be accountable for all aspects of the work: RV, JM, DV, BS.

### Conflict of interest statement

The authors declare that the research was conducted in the absence of any commercial or financial relationships that could be construed as a potential conflict of interest.

## References

[B1] Akerlund E. Esbjornsson E. Sunnerhagen K. S. Björkdahl A. (2013). Can computerized working memory training improve impaired working memory, cognition and psychological health? Brain Inj. 27, 1649–1657. 10.3109/02699052.2013.830195 24087909

[B2] Anguera J. A. Boccanfuso J. Rintoul J. L. Al-Hashimi O. Faraji F. Janowich J.. (2013). Video game training enhances cognitive control in older adults. Nature 501, 97. 10.1038/nature12486 24005416 PMC3983066

[B3] Baddeley A. (1992). Working memory. Science 255, 556–559. 10.1126/science.1736359 1736359

[B4] Bender R. Lange S. (2001). Adjusting for multiple testing - when and how? J. Clin. Epidemiol. 54, 343–349. 10.1016/S0895-4356(00)00314-0 11297884

[B5] Björkdahl A. Akerlund E. Svensson S. Esbjornsson E. (2013). A randomized study of computerized working memory training and effects on functioning in everyday life for patients with brain injury. Brain Inj. 27, 1658–1665. 10.3109/02699052.2013.830196 24131298

[B6] Chen S. H. Thomas J. D. Glueckauf R. L. Bracy O. L. (1997). The effectiveness of computer-assisted cognitive rehabilitation for persons with traumatic brain injury. Brain Inj. 11, 197–209. 10.1080/026990597123647 9058001

[B7] Cicerone K. D. Langenbahn D. M. Braden C. Malec J. F. Kalmar K. Fraas M. Ashman T. (2011). Evidence-based cognitive rehabilitation: Updated review of the literature from 2003 through 2008. Arch. Phys. Med. Rehabil., 92, 519–530. 10.1016/j.apmr.2010.11.015 21440699

[B8] Corbett A. Owen A. Hampshire A. Grahn J. Stenton R. Dajani S.. (2015). The effect of an online cognitive training package in healthy older adults: an online randomized controlled trial. J. Am. Med. Dir. Assoc. 16, 990–997. 10.1016/j.jamda.2015.06.014 26543007

[B9] Cruz V. T. Pais J. Alves I. Ruano L. Mateus C. Barreto R.. (2014). Web-based cognitive training: patient adherence and intensity of treatment in an outpatient memory clinic. J. Med. Internet Res. 16, 130–140. 10.2196/jmir.3377 24808451 PMC4034117

[B10] Cumming T. B. Marshall R. S. Lazar R. M. (2013). Stroke, cognitive deficits, and rehabilitation: still an incomplete picture. Int. J. Stroke 8, 38–45. 10.1111/j.1747-4949.2012.00972.x 23280268

[B11] Curran-Everett D. (2000). Multiple comparisons: philosophies and illustrations. Am. J. Physiol. Regul. Integr. Comp. Physiol. 279, R1–R8. 10896857 10.1152/ajpregu.2000.279.1.R1

[B12] del Ser T. Barba R. Morin M. M. Domingo J. Cemillan C. Pondal M.. (2005). Evolution of cognitive impairment after stroke and risk factors for delayed progression. Stroke 36, 2670–2675. 10.1161/01.STR.0000189626.71033.35 16254227

[B13] De Luca R. Calabro R. S. Gervasi G. De Salvo S. Bonanno L. Corallo F.. (2014). Is computer-assisted training effective in improving rehabilitative outcomes after brain injury? A case-control hospital-based study. Dis. Health J. 7, 356–360. 10.1016/j.dhjo.2014.04.003 24947578

[B14] Desmond D. Moroney J. Sano M. Stern Y. (1996). Recovery of cognitive function after stroke. Stroke 27, 1798–1803. 10.1161/01.STR.27.10.1798 8841333

[B15] Dugard P. File P. Todman J. (2011). Single-Case and Small-N Experimental Designs: A Practical Guide to Randomization Tests, 2nd Edn. Hove; Sussex; New York: Routledge.

[B16] Fernandez E. Bringas M. L. Salazar S. Rodriguez D. Garcia M. E. Torres M. (2012). Clinical impact of RehaCom software for cognitive rehabilitation of patients with acquired brain injury. MEDICC Rev. 14, 32–35. 10.1590/S1555-79602012000400007 23154316

[B17] Gauggel S. Niemann T. (1996). Evaluation of a short-term computer-assisted training programme for the remediation of attentional deficits after brain injury: a preliminary study. Int. J. Rehabil. Res. 19, 229–239. 10.1097/00004356-199609000-00004 8910125

[B18] Glickman M. E. Rao S. R. Schultz M. R. (2014). False discovery rate control is a recommended alternative to bonferroni-type adjustments in health studies. J. Clin. Epidemiol. 67, 850–857. 10.1016/j.jclinepi.2014.03.012 24831050

[B19] Gray J. M. Robertson I. Pentland B. Anderson S. (1992). Microcomputer-based attentional retraining after brain damage: a randomised group controlled trial. Neuropsychol. Rehabil. 2, 97–115. 10.1080/09602019208401399

[B20] Gronwall D. M. A. (1977). Paced auditory serial-addition task: measure of recovery from concussion. Percept. Mot. Skills 44, 367–373. 10.2466/pms.1977.44.2.367 866038

[B21] Hamzei F. Liepert J. Dettmers C. Weiller C. Rijntjes M. (2006). Two different reorganization patterns after rehabilitative therapy: an exploratory study with fMRI and TMS. Neuroimage 31, 710–720. 10.1016/j.neuroimage.2005.12.035 16516499

[B22] Hauke J. Fimm B. Sturm W. (2011). Efficacy of alertness training in a case of brainstem encephalitis: clinical and theoretical implications. Neuropsychol. Rehabil. 21, 164–182. 10.1080/09602011.2010.541792 21391120

[B23] Kelly C. Foxe J. J. Garavan H. (2006). Patterns of normal human brain plasticity after practice and their implications for neurorehabilitation. Arch. Phys. Med. Rehabil. 87, S20–S29. 10.1016/j.apmr.2006.08.333 17140876

[B24] Kimberley T. J. Samargia S. Moore L. G. Shakya J. K. Lang C. E. (2010). Comparison of amounts and types of practice during rehabilitation for traumatic brain injury and stroke. J. Rehabil. Res. Dev. 47, 851–861. 10.1682/JRRD.2010.02.0019 21174250

[B25] Kurland J. Baldwin K. Tauer C. (2010). Treatment-induced neuroplasticity following intensive naming therapy in a case of chronic wernicke's aphasia. Aphasiology 24, 737–751. 10.1080/02687030903524711

[B26] Lampit A. Hallock H. Valenzuela M. (2014). Computerized cognitive training in cognitively healthy older adults: a systematic review and meta-analysis of effect modifiers. PLoS Med. 11:e1001756. 10.1371/journal.pmed.1001756 25405755 PMC4236015

[B27] Larson E. B. Feigon M. Gagliardo P. Dvorkin A. Y. (2014). Virtual reality and cognitive rehabilitation: a review of current outcome research. NeuroRehabilitation 34, 759–772. 10.3233/NRE-141078 24820166

[B28] Lin Z. Tao J. Gao Y. Yin D. Chen A. Chen L. (2014). Analysis of central mechanism of cognitive training on cognitive impairment after stroke: Resting-state functional magnetic resonance imaging study. J. Int. Med. Res. 42, 659–668. 10.1177/0300060513505809 24722262

[B29] Lundqvist A. Grundstrom K. Samuelsson K. Ronnberg J. (2010). Computerized training of working memory in a group of patients suffering from acquired brain injury. Brain Inj. 24, 1173–1183. 10.3109/02699052.2010.498007 20715888

[B30] Maaijwee N. A. M. M. Schaapsmeerders P. Rutten-Jacobs L. C. A. Arntz R. M. Schoonderwaldt H. C. van Dijk E. J. (2014). Subjective cognitive failures after stroke in young adults: prevalent but not related to cognitive impairment. J. Neurol. 261, 1300–1308. 10.1007/s00415-014-7346-3 24740819

[B31] Man D. W. K. Soong W. Y. L. Tam S. F. HuiChan C. W. Y. (2006). Self-efficacy outcomes of people with brain injury in cognitive skill training using different types of trainer-trainee interaction. Brain Inj. 20, 959–970. 10.1080/02699050600909789 17062427

[B32] Middleton L. E. Lam B. Fahmi H. Black S. E. McIlroy W. E. Stuss D. T.. (2014). Frequency of domain-specific cognitive impairment in sub-acute and chronic stroke. NeuroRehabilitation 34, 305–312. 24401826 10.3233/NRE-131030

[B33] Miyake A. Friedman N. P. Emerson M. J. Witzki A. H. Howerter A. Wager T. D. (2000). The unity and diversity of executive functions and their contributions to complex “frontal lobe” tasks: a latent variable analysis. Cogn. Psychol. 41, 49–100. 10.1006/cogp.1999.0734 10945922

[B34] Moher D. Liberati A. Tetzlaff J. Altman D. G. The PRISMA Group (2009). Preferred reporting items for systematic reviews and meta-analyses: The PRISMA Statement. PLoS Med. 6:e1000097. 10.1371/journal.pmed1000097 19621072 PMC2707599

[B35] Nordvik J. E. Schanke A. Walhovd K. Fjell A. Grydeland H. Landro N. I. (2012). Exploring the relationship between white matter microstructure and working memory functioning following stroke: a single case study of computerized cognitive training. Neurocase 18, 139–151. 10.1080/13554794.2011.568501 21780988

[B36] Nordvik J. E. Walle K. M. Nyberg C. K. Fjell A. M. Walhovd K. B. Westlye L. T.. (2014). Bridging the gap between clinical neuroscience and cognitive rehabilitation: The role of cognitive training, models of neuroplasticity and advanced neuroimaging in future brain injury rehabilitation. NeuroRehabilitation 34, 81–85. 10.3233/NRE-131017 24284460

[B37] Owen A. M. Hampshire A. Grahn J. A. Stenton R. Dajani S. Burns A. S.. (2010). Putting brain training to the test. Nature 465. 775–778. 10.1038/nature09042 20407435 PMC2884087

[B38] Park N. W. Ingles J. L. (2001). Effectiveness of attention rehabilitation after an acquired brain injury: a meta-analysis. Neuropsychology 15, 199–210. 10.1037/0894-4105.15.2.199 11324863

[B39] Ponsford J. L. Kinsella G. (1988). Evaluation of a remedial programme for attentional deficits following closed-head injury. J. Clin. Exp. Neuropsychol. 10, 693–708. 10.1080/01688638808402808 3235646

[B40] Poulin V. Korner-Bitensky N. Dawson D. R. Bherer L. (2012). Efficacy of executive function interventions after stroke: a systematic review. Top. Stroke Rehabil. 19, 158–171. 10.1310/tsr1902-158 22436364

[B41] Prokopenko S. V. Mozheyko E. Y. Petrova M. M. Koryagina T. D. Kaskaeva D. S. Chernykh T. V.. (2013). Correction of post-stroke cognitive impairments using computer programs. J. Neurol. Sci. 325, 148–153. 10.1016/j.jns.2012.12.024 23312291

[B42] Robertson I. H. Murre J. M. J. (1999). Rehabilitation of brain damage: brain plasticity and principles of guided recovery. Psychol. Bull. 125, 544–575. 10.1037/0033-2909.125.5.544 10489541

[B43] Rothman K. J. (1990). No adjustments are needed for multiple comparisons. Epidemiology 1, 43–46. 10.1097/00001648-199001000-00010 2081237

[B44] Ruff R. M. Mahaffey R. Engel J. Farrow C. Cox D. Karzmark P. (1994). Efficacy study of THINKable in the attention and memory retraining of traumatically head-injured patients. Brain Inj. 8, 3–14. 10.3109/02699059409150954 8124315

[B45] Spikman J. M. Boelen D. H. E. Lamberts K. F. Brouwer W. H. Fasotti L. (2010). Effects of a multifaceted treatment program for executive dysfunction after acquired brain injury on indications of executive functioning in daily life. J. Int. Neuropsychol. Soc. 16, 118–129. 10.1017/S1355617709991020 19900348

[B46] Sturm W. Fimm B. Cantagallo A. Cremel N. North P. Passadori A. (2003). Specific computerized attention training in stroke and traumatic brain-injured patients: a european multicenter efficacy study. Zeitschrift Fur Neuropsychol. 14, 283–292. 10.1024/1016-264X.14.4.283

[B47] Sturm W. Willmes K. Orgass B. Hartje W. (1997). Do specific attention deficits need specific training? Neuropsychol. Rehabil. 7, 81–103. 10.1080/713755526

[B48] Takeuchi N. Izumi S. (2015). Combinations of stroke neurorehabilitation to facilitate motor recovery: perspectives on hebbian plasticity and homeostatic metaplasticity. Front. Hum. Neurosci. 9:349. 10.3389/fnhum.2015.00349 26157374 PMC4477170

[B49] Tham W. Auchus A. P. Thong M. Goh M. L. Chang H. M. Wong M. C.. (2002). Progression of cognitive impairment after stroke: one year results from a longitudinal study of singaporean stroke patients. J. Neurol. Sci. 203, 49–52. 10.1016/S0022-510X(02)00260-5 12417356

[B50] Thrane G. Friborg O. Anke A. Indredayik B. (2014). A meta-analysis of constraint-induced movement therapy after stroke. J. Rehabil. Med. 46, 833–842. 10.2340/16501977-1859 25182341

[B51] Toril P. Reales J. M. Ballesteros S. (2014). Video game training enhances cognition of older adults: a meta-analytic study. Psychol. Aging 29, 706–716. 10.1037/a0037507 25244488

[B52] van Vleet T. M. Chen A. Vernon A. Novakovic-Agopian T. D'Esposito M. T. (2015). Tonic and phasic alertness training: a novel treatment for executive control dysfunction following mild traumatic brain injury. Neurocase 21, 489–498. 10.1080/13554794.2014.928329 24984231

[B53] Westerberg H. Jacobaeus H. Hirvikoski T. Clevberger P. Ostensson M. L. Bartfai A.. (2007). Computerized working memory training after stroke - A pilot study. Brain Inj. 21, 21–29. 10.1080/02699050601148726 17364516

[B54] Zickefoose S. Hux K. Brown J. Wulf K. (2013). Let the games begin: a preliminary study using attention process training-3 and LumosityTM brain games to remediate attention deficits following traumatic brain injury. Brain Inj. 27, 707–716. 10.3109/02699052.2013.775484 23672446

