# Supplementary material for: Computer-Based Cognitive Training for Executive Functions after Stroke: A Systematic Review
Source: Front Hum Neurosci. 2016 Apr 20;10:150. doi: 10.3389/fnhum.2016.00150 (PMC4837156; doi:10.3389/fnhum.2016.00150)
Supplement: Supplementary file 1 [file DataSheet1.PDF]

Supplementary Material

Computer-Based Cognitive Training for Executive Functions after Stroke: A Systematic Review

Renate M. van de Ven*, Jaap M. J. Murre, Dick J. Veltman, Ben A. Schmand
*** Correspondence:** Corresponding Author: r.m.vandeven@uva.nl

Supplement 1 Search strategy

The search strategy was created by Renate van de Ven, Janneke Staaks, and Ben Schmand.

Search Question

Cognitive training of executive functioning, attention, and working memory. Training aim needs to be restoration, thus not improvement of internal or external strategies (e.g., excluding PDA). Training needs to be done on a computer, but not virtual reality or simulations. Study aim should not merely be feasibility of a training but also includes outcome measures.

Databases

PsycINFO	320 results (May 12, 2015)
Medline	958 results (May 12, 2015)
Web of Science	546 results (May 12, 2015)
Cochrane Library	176 results (May 12, 2015)
**Total**	**2000**
**Total, deduplicated**	1469

PsycINFO
Ovid

#1 stroke or acquired brain injury, adults

cerebrovascular accidents/ OR traumatic brain injury/ OR (brain injur* OR brain damage* OR poststroke* OR stroke OR strokes).ti,ab,id.
Results: 53.108 (May 12, 2015)

#2a computer training

computer training/ OR brain game*.ti,ab,id. OR (((computer assisted instruction/ OR computer assisted therapy/ OR computer games/ OR computers/ OR computer software/ OR internet/ OR websites/ OR (computer* OR digital* OR electronic OR gaming OR internet* OR video game* OR online OR on-line OR website* OR web).ti,ab,id.) AND (memory training/ OR training/ OR (enhancement OR restor* OR restitut* OR retrain* OR training* OR treatment*).ti,ab,id.)))
Results: 28.877 (May 12, 2015)

#3 executive functioning

attention/ OR cognition/ OR cognitive ability/ OR cognitive control/ OR cognitive processing speed/ OR executive function/ OR set shifting/ or short term memory/ OR task switching/ OR (attention* OR cognit* OR executive function* OR memory OR neuropsychological* OR processing speed* OR set shifting OR task switch* OR switchcost* OR switch cost*).ti,ab,id,tm.
Results: 648.305 (May 12, 2015)

#4 Animals

exp animals/

(1 AND 2a AND 3) NOT 4 297 results (May 12, 2015)
Addition head injur*: +23
PsycINFO reference set (8 van de 11)
(2013-41749-021 OR 2013-41749-022 OR 2014-25428-017 OR 2010-17392-003 OR 2012-05269-008 OR 2013-02946-012 OR 2007-03094-003 OR 2013-17202-010).an.

Medline
Ovid MEDLINE In-Process & Other Non-Indexed Citations and Ovid MEDLINE

#1 stroke or acquired brain injury, adults
brain concussion/ OR brain hemorrhage, traumatic/ OR brain infarction/ OR brain injuries/ OR brain injury, chronic/ OR brain stem infarctions/ OR diffuse axonal injury/ OR infarction, anterior cerebral artery/ OR infarction, middle cerebral artery/ OR infarction, posterior cerebral artery/ OR stroke/ OR stroke, lacunar/ OR (brain injur* OR brain damage* OR poststroke* OR stroke OR strokes).ti,ab,kf.
Results: 254.260 (May 12, 2015)

#2 computer training
brain game*.ti,ab,kf. OR ((computer-assisted instruction/ OR internet/ OR software/ OR therapy, computer-assisted/ OR user-computer interface/ OR video games/ OR (computer* OR digital* OR electronic OR gaming OR internet* OR video game* OR online OR on-line OR website* OR web).ti,ab,kf.) AND (enhancement OR restor* OR restitut* OR retrain* OR training* OR treatment*).ti,ab,kf.)
Results: 112.480 (May 12, 2015)

#3 executive functioning
attention/ OR cognition/ OR cognition disorders/ OR executive function/ OR learning/ OR memory disorders/ OR memory, short term/ OR neuropsychological tests/ OR treatment outcome/ OR reaction time/ OR (attention* OR cognit* OR executive function* OR memory OR neuropsychological* OR processing speed* OR set shifting OR task switch* OR switchcost* OR switch cost*).ti,ab,kf.
Results: 1.411.054 (May 12, 2015)

#4 Animals
animals/

(1 AND 2a AND 3) NOT 4 933 results (May 12, 2015)

Addition head injur*: + 25

Medline reference set (10 van de 11)
(24087909 OR 24131298 OR 24947578 OR 23154316 OR 21843045 OR 20715888 OR 21780988 OR 23312291 OR 17364516 OR 23672446).ui.

Web of Science
Web of Science Core Collection

#1 stroke or acquired brain injury, adults
TS=("brain injur*" OR "brain damage*" OR "poststroke*" OR "stroke" OR "strokes")
Results: 270.803 (May 12, 2015)

#2 computer training
TS=("brain game*" OR (("computer*" OR "digital*" OR "electronic" OR "gaming" OR "internet*" OR "video game*" OR "online" OR "on-line" OR "website*" OR "web") AND ("enhancement" OR "restor*" OR "restitut*" OR "retrain*" OR "training*" OR "treatment*")))
Results: 144.048 (May 12, 2015)

#3 executive functioning
TS=("attention*" OR "cognit*" OR "executive function*" OR "memory" OR "neuropsychological*" OR "processing speed" OR "set shifting" OR "task switch*" OR "switchcost*" OR "switch cost*")
Results: 1.055.704 (May 12, 2015)

(1 AND 2 AND 3) 527 results (May 12, 2015)

Addition head injur*: +19

Cochrane Library
Wiley Online Library

#1 stroke or acquired brain injury, adults
"brain injur*":ti,ab,kw OR "brain damage*":ti,ab,kw OR "head injur*":ti,ab,kw OR "poststroke*":ti,ab,kw OR "stroke":ti,ab,kw OR "strokes":ti,ab,kw
Results: 28.941 (May 12, 2015)

#2 computer training
"brain game*":ti,ab,kw OR (("computer*":ti,ab,kw OR "digital*":ti,ab,kw OR "electronic":ti,ab,kw OR "gaming":ti,ab,kw OR "internet*":ti,ab,kw OR "video game*":ti,ab,kw OR "online":ti,ab,kw OR "on-line":ti,ab,kw OR "website*":ti,ab,kw OR "web":ti,ab,kw) AND ("enhancement":ti,ab,kw OR "restor*":ti,ab,kw OR "restitut*":ti,ab,kw OR "retrain*":ti,ab,kw OR "training*":ti,ab,kw OR "treatment*":ti,ab,kw))
Results: 17.115 (May 12, 2015)

#3 executive functioning
"attention*":ti,ab,kw OR "cognit*":ti,ab,kw OR "executive function*":ti,ab,kw OR "memory":ti,ab,kw OR "neuropsychological*":ti,ab,kw OR "processing speed":ti,ab,kw OR "set shifting":ti,ab,kw OR "task switch*":ti,ab,kw OR "switchcost*":ti,ab,kw OR "switch cost*":ti,ab,kw
Results: 46.767 (May 12, 2015)

1 AND 2 AND 3 170 results (May 12, 2015)

Addition head injur*: +6 results
